# Self‐Assembled Peptide‐Gold Nanoclusters with SiRNA Targeting Telomeric Response to Enhance Radiosensitivity in Lung Cancer Cells

**DOI:** 10.1002/smsc.202400156

**Published:** 2024-12-16

**Authors:** Sean Moro, Mohammed Omrani, Sule Erbek, Muriel Jourdan, Catharina I. Vandekerckhove, Cyril Nogier, Laetitia Vanwonterghem, Marie‐Carmen Molina, Pau Bernadó, Aurélien Thureau, Jean‐Luc Coll, Olivier Renaudet, Xavier Le Guével, Virginie Faure

**Affiliations:** ^1^ Institute for Advanced Biosciences University Grenoble Alpes INSERM U1209 CNRS UMR 5309 38000 Grenoble France; ^2^ Department of Molecular Chemistry (DCM) University Grenoble Alpes CNRS DCM UMR 5250 F‐38000 Grenoble France; ^3^ Department of Molecular Pharmacochemistry University Grenoble Alpes CNRS UMR5063 ICMG FR 2607 38000 Grenoble France; ^4^ Centre de Biologie Structurale (CBS) Université Montpellier INSERM CNRS. 29 rue de Navacelles 34090 Montpellier France; ^5^ Swing Beamline Synchrotron SOLEIL 91192 Gif sur Yvette France

**Keywords:** cancers, gold nanoclusters, radiotherapies, siRNA, telomeres

## Abstract

Lung cancer cells resistant to radiotherapy present a significant clinical challenge. Stable telomeric structures, maintained by the TRF2 protein, play a critical role in protecting cells from ionizing radiation. Reduced TRF2 expression increases DNA damage and radiosensitivity. We designed a self‐assembling system utilizing ultra‐small luminescent gold nanoclusters (AuNCs) with radiosensitizing properties, combined with siRNA targeting TRF2. The system forms ≈100  nm non‐spherical structures with AuNCs enriched in the outer layer, exhibiting a 17.6‐fold enhancement in red photoluminescence due to aggregation‐induced effects. This nanoplatform efficiently penetrates lung cancer cells, reducing TRF2 expression by 50%. Under 5 Gy radiotherapy, cells treated with this system show a 1.5‐fold radiosensitivity increase from AuNCs and a 2.3‐fold reduction in clonogenic survival due to telomere deprotection. The AuNC‐siRNATRF2 system combines enhanced optical properties with biological functionality, offering a promising approach to augment radiotherapy efficacy by disrupting telomeric protective mechanisms in cancer cells.

## Graphical abstract

Lung cancer cells resistant to radiotherapy pose a challenge, linked to TRF2 protein's role in telomere protection. A self‐assembling system with luminescent gold nanoclusters and siRNA downregulates TRF2, enhancing radiosensitivity. This 100 nm nonspherical structure boosts photoluminescence and penetrates cells, reducing TRF2 expression by 50% and clonogenic survival by 2.3‐fold, promising improved radiotherapy.

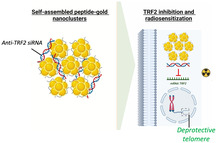

## 1 Introduction

Nonsmall cell lung cancer (NSCLC), one of the most commonly diagnosed cancers, is the leading cause of cancer‐related death worldwide.^[^ ^1^ ^]^ Although radiotherapy (RT) is reckoned as an important and effective treatment option for NSCLC, some patients present rapid progression and resistance to RT and eventually relapse shortly after treatment ends.^[^ ^2^ ^]^ Consequently, the development of novel radiosensitizing strategies with low side effects in the treatment of NSCLC is urgently required to prolong the survival and the quality of life of patients.

Among the different mechanisms involved in the regulation of cellular radiosensitivity/radioresistance, the adaptative telomeric response has been highlighted in an increasing number of studies.^[^ ^3^ ^]^ Telomeres are nucleoprotein structures protecting the extremities of eukaryotic linear chromosomes from DNA instability. A telomere‐specific protein complex, termed shelterin, has a crucial function in safeguarding and securing telomere integrity by maintaining a T‐loop telomeric structure that protects telomeres. One of the main factors of this complex is the Telomeric Repeat binding Factor 2 (TRF2). It has been demonstrated that partial suppression of TRF2 by siRNA (small interfering RNA) in mesenchymal stem cells increased DNA damage and senescence and reduced the surviving fraction in response to ionizing radiations (IR).^[^ ^4^ ^]^ TRF2 was also described as a radioresistant protein in human lung carcinoma cells.^[^ ^5^ ^]^ Therefore, the development of anti‐TRF2 compounds in combination with IR may represent a valuable approach to enhance the effect of RT in NSCLC and reduce radiation dose delivered to patients, thus minimizing side effects in normal tissue.

The small interfering (si)RNA‐based therapy has emerged as a promising strategy to downregulate a targeted protein as TRF2. However, the efficiency of gene silencing by naked siRNA is very low, because these molecules are rapidly degraded by nucleases in the bloodstream and experience rapid renal clearance in the body. Furthermore, the negative charges of siRNAs hamper their penetration across the cell membrane and prevent its intracellular accumulation. Therefore, it is necessary to both protect siRNAs from degradation and to deliver them into the cytoplasm of the targeted cells.

Gold nanoparticles (AuNPs) are widely recognized to be a reliable siRNA delivery platform due to their ratio of high surface area/volume and their versatile tunable surface chemistry.^[^ ^6^ ^]^ They are biocompatible and have the capacity to protect siRNA against degradation. In addition, thanks to their high atomic number (*Z* = 79), AuNPs have a large X‐ray absorption cross section, which makes them good radiosensitizers to improve the effectiveness of RT. Indeed, Hainfeld et al. were the first to demonstrate an increased radiotherapeutic efficacy by the presence of AuNPs with a fourfold improvement in survival rate in syngeneic mouse models with subcutaneous breast cancer.^[^ ^7^ ^]^ In addition, a reduction in tumor mass has been observed after RT using very small AuNPs (<5 nm) in mice bearing subcutaneous ovarian cancer. Therefore, AuNPs may improve the effectiveness of radiation therapy by increasing the tumor's sensitivity to radiation and giving the possibility of reducing the radiation dose delivered. Another notable advantage of AuNPs lies in their ability to penetrate and passively accumulate in tumors through an enhanced permeability and retention effect (EPR). This phenomenon results in substantially higher local concentrations of nanoparticles at the tumor site, often reaching concentrations 10 to 50 times greater than those found in normal tissue.^[^ ^7^, ^8^, ^9^ ^]^

We are particularly interested in a subfamily of AuNPs with ultrasmall size less than 3 nm called gold nanoclusters (AuNCs). Recent works have demonstrated that these theranostic agents are well suited for the monitoring and treatment of cancers.^[^ ^10^, ^11^, ^12^, ^13^ ^]^ Unlike AuNPs, these “nanoprobes” exhibit molecular‐like properties, such as photoluminescence in near‐infrared (NIR) optical window (700–900 nm)^[^ ^14^ ^]^ and in the shortwave infrared (SWIR) optical window (900–1700 nm),^[^ ^15^, ^16^, ^17^ ^]^ allowing noninvasive tracking on mice models by optical fluorescence imaging.^[^ ^18^, ^19^, ^20^ ^]^ Due to their ultrasmall size below 5 nm, AuNCs present a good compromise between an accumulation in the tumor microenvironment and efficient renal elimination.^[^ ^21^ ^]^ We and others have also reported an increased absorption and photoluminescence of self‐assembled AuNCs,^[^ ^22^, ^23^ ^]^ which can be exploited not only to improve their detection at tumor sites but also to protect and efficiently deliver biomolecules such as nucleotides.^[^ ^24^ ^]^ Thus, AuNCs represent an interesting delivery platform that could be metabolized by cells and cleared efficiently by the kidneys, which should mitigate toxicity resulting from prolonged storage in various organs.^[^ ^25^, ^26^, ^27^, ^28^ ^]^

**Figure 1:**
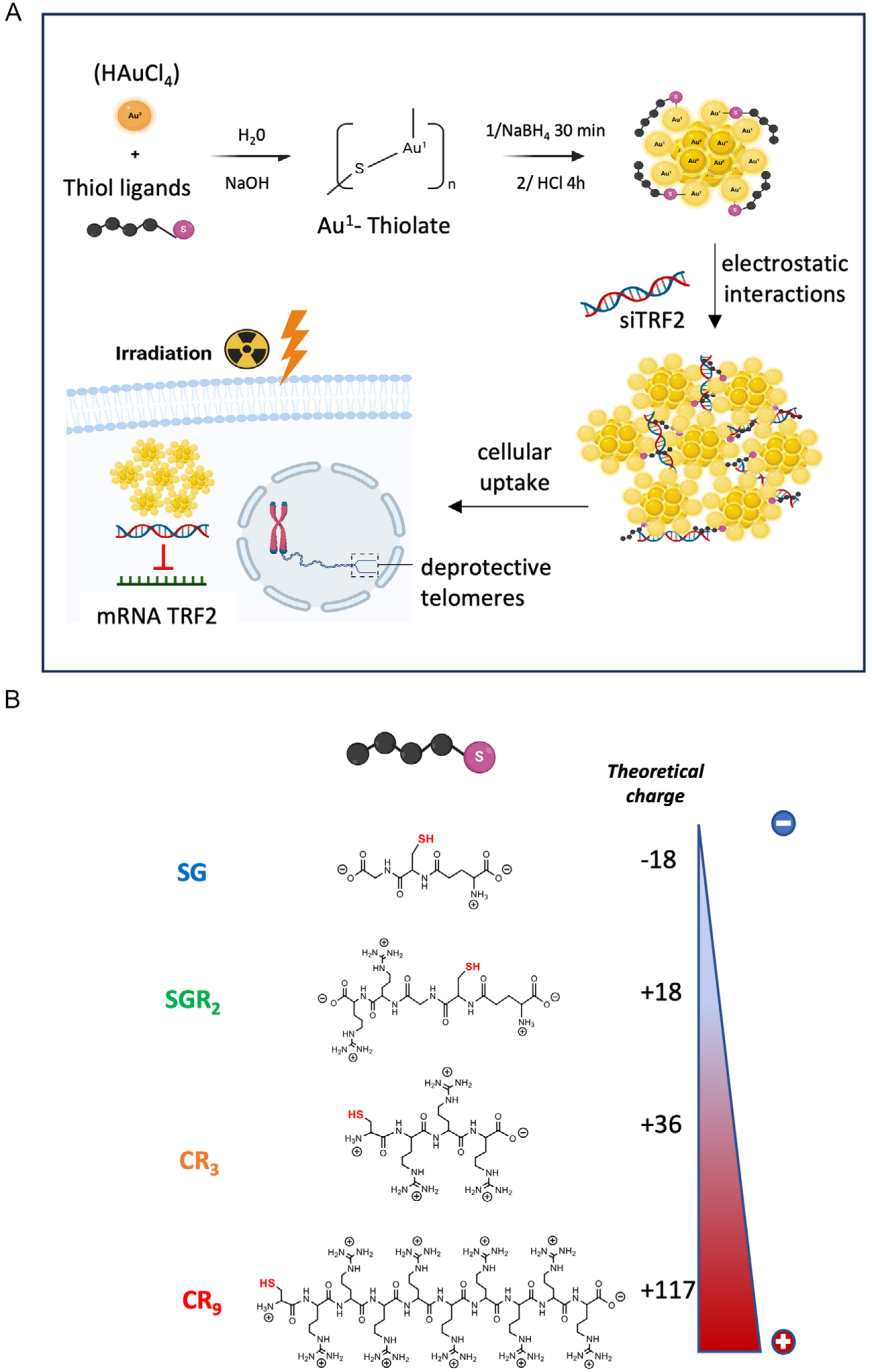
Synthesis, self‐assembly, and cellular delivery of AuNC‐siRNA for telomeric response targeting to enhance the impact of irradiation on cells. A) Positively charged AuNCs in the presence of siRNA form self‐assemblies. After cellular uptake of AuNCs‐siRNA, siRNA is delivered within the cytoplasm to knock down the telomeric protein TRF2. The radiosensitizing proprieties of AuNCs as well as the telomeric response targeting would improve radiation effects. B) Thiolated peptide ligands (SG: glutathione, SGR_2_: glutathione with 2 arginines, CR_3_: tetrapeptide with 25% of SG, CR_9_: decapeptide with 25% of SG) and their different global charge when stabilizing AuNCs.

In this study, we have developed a multimodal cancer therapy based on the development of new radiosensitizer AuNCs‐nanosystems able to deliver siRNA targeting the telomeric response to potentiate radiation therapy in lung cancer cells (**Figure** **1**A). Interestingly, these monodisperse NIR‐emitting self‐assemblies showed an original organization with a compaction of siRNA displayed by AuNCs mainly on the outer layer. We have evaluated the synergic effect between the deprotection of telomeres and the radiosensitizing effect of AuNCs.

## 2 Results and Discussion

### 2.1 Physicochemical Characterization and Morphology of AuNCs

The AuNCs were synthesized using wet chemistry following a standard method with the complexation and the nucleation of gold nanoclusters in aqueous solution^[^ ^29^ ^]^ (Figure 1A). We used peptides with increasing positive charge achieved by adjusting the number of arginine groups: SG < SGR_2_ < CR_3_ < CR_9_. (Figure 1B). To increase the number of arginine (R) per peptide, we moved from a modified‐glutathione with arginine (SGR_2_)^[^ ^30^ ^]^ to multiple arginines associated with a cysteine (C) (CR_3_, CR_9_) to obtain stable AuNCs. High‐resolution transmission electron microscopy (HRTEM) images revealed ultrasmall sizes ranging from 2.1 to 2.8 nm for all nanoclusters, regardless of the nature of the ligand (Figure S1, Supporting Information, **Table** **1**). AuNCs exhibit excellent solubility in both water and PBS buffer, thanks to the water‐soluble peptide that stabilizes the gold core.

**Table 1:** Physicochemical properties of AuNCs and self‐assembled AuNCs‐siRNA. Surface charge determined by zeta potential at pH 7, gold core size measured by HRTEM on average of 100 particles. Hydrodynamic diameters are measured by NMR DOSY for AuNCs (in deuterated water) at 25 °C and by DLS for AuNCs‐siRNA in deionized water.

Au NCs	Mean Zeta potential (mV)	Core size by TEM (nm)	Hydrodynamic diameter (nm)
AuSG	−16.7 ± 2.0	2.1 ± 0.3	1.66 ± 0.01a) (DOSY)
AuSGR_2_	+5.4 ± 1.7	2.3 ± 0.3	2.4 ± 0.2 (DOSY)
AuCR_3_	+11.1 ± 1.5	2.3 ± 0.6	2.2 ± 0.2 (DOSY)
AuCR_9_	+20.1 ± 2.1	2.8 ± 0.5	2.2 ± 0.2 (DOSY)
AuSGR_2_ + siRNA	+14.0 ± 0.3	112 ± 27	122 ± 42 (DLS)
AuCR_3_ + siRNA	+16.0 ± 0.5	107 ± 13	142 ± 33 (DLS)
AuCR_9_ + siRNA	+23.5 ± 0.3	107 ± 30	120 ± 80 (DLS)

a) Data obtained from Journal of Physical Chemistry C, 123 (2019) 26705–26717.

We determined the hydrodynamic diameter of these AuNCs in D_2_O using NMR diffusion‐ordered spectroscopy (NMR‐DOSY), which offers high resolution and prevents artifacts compared to dynamic light scattering (DLS) for such small particle sizes. We observed an increase in the hydrodynamic diameter of AuNCs with the size of the ligand, ranging from 1.66 ± 0.01 nm for AuSG to 2.2 ± 0.2 nm for AuCR_9_ (Table 1, Figure S2, Supporting Information). Zeta potential measurements conducted in water at pH 7 demonstrated a consistent increase in surface charge, with AuSG (−16.7 ± 2.0 mV) <AuSGR_2_ (+5.4 ± 1.7 mV) < AuCR_3_ (+11.1 ± 1.5 mV) < AuCR_9_ (+20.1 ± 2.1 mV). This trend aligns with the expected increase in arginine content per peptide, resulting in a theoretical charge ranging from −1 for SG to +9 for CR_9_ at pH 7 (Figure S3, Supporting Information).

Assuming an average of 18 ligands per AuNC for these types of AuNCs,^[^ ^30^ ^]^ we estimated a theoretical charge of −18 for AuSG and +117 for AuCR_9_ (Figure 1B and Figure S4, Supporting Information). Experimental values for AuCR_3_ and AuCR_9_ (Table 1) were found to be lower than the theoretical charge, leading us to hypothesize that the positively charged arginine may be involved in strong electrostatic interactions with the negative groups of the ligand or with the gold surface, thereby reducing the overall surface charge.^[^ ^30^, ^31^ ^]^ The positive charge of AuNCs at pH 7 was further confirmed through agarose gel electrophoresis, revealing a fluorescent band for AuSGR_2_, AuCR_3_, and AuCR_9_ on the anode side, contrasting with the band observed for AuSG on the cathode side (Figure S5, Supporting Information). Overall, these results indicated the ability to design ultrasmall peptide‐AuNCs with a controlled positive surface charge at pH 7.

### 2.2 Physicochemical Characterization and Morphology of Self‐Assembled AuNCs‐siRNA

Self‐assembled AuNCs with siRNA were obtained through electrostatic interactions between positively charged AuNCs (AuSGR_2_, AuCR_3_, AuCR_9_) and siRNA_TRF2_, which is negatively charged.

**Figure 2:**
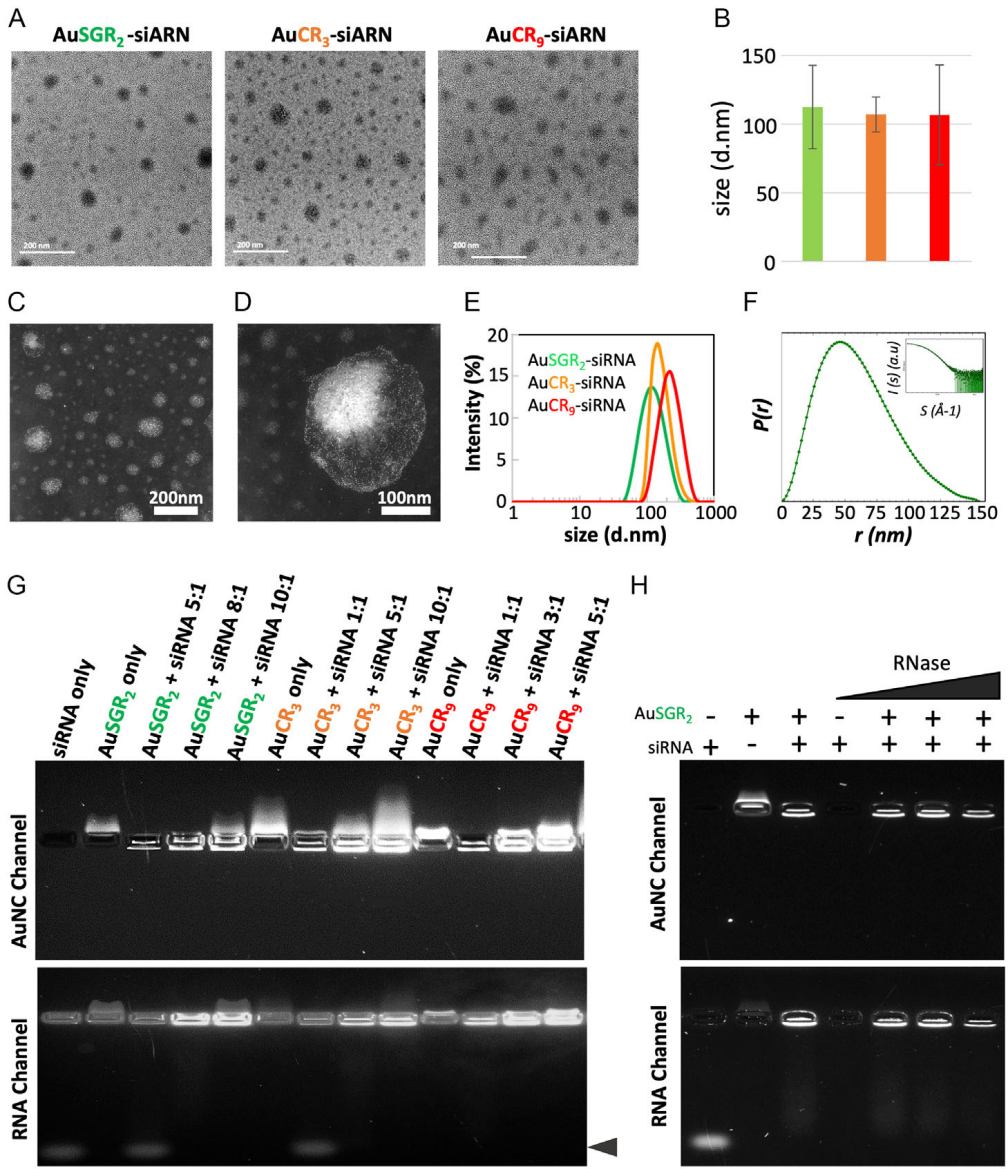
Physicochemical properties of self‐assembled AuNCs‐siRNA. A) TEM (Transmission Electron Microscopy) images of the self‐assembled AuNCs‐siRNA: AuSGR_2_‐siRNA, AuCR_3_‐siRNA, and AuCR_9_‐siRNA. B) Average size distribution of the self‐assembled AuNCs‐siRNA determined on TEM images using at least 100 particles. C,D) STEM images of the self‐assembled AuSGR_2‐_siRNA. E) Hydrodynamic diameter of self‐assembled AuNCs‐siRNA in aqueous solution. F) Pair‐wise distance distribution, *P*(*r*), obtained from the Fourier transform of the SAXS curve (inset) measured for a 1/10 dilution of the self‐assembled AuSGR_2_‐siRNA in PBS 1X buffer. G) Analysis of the formation of AuNCs‐siRNA between siRNA_TRF2_ and AuSGR_2_ or AuCR_3_ or AuCR_9_ by electrophoresis in 4% agarose gel using the fluorescent channel of AuNCs (up) and after siRNA staining with GoldSyber (down) at different weight ratio (AuNC:siRNA). H) Migration of AuNCs and self‐assembled AuNCs‐siRNA in agarose gel in the presence of increasing concentrations of RNase (1, 5, and 10 ng μL^−1^). AuSGR_2_ was detected using the fluorescent channel of AuNCs (up) and after siRNA staining (down). The arrow indicates free siRNA.

HRTEM images of the self‐assembled AuNCs with siRNA_TRF2_ obtained with the three types of AuNCs showed an average size of around 110 nm (Table 1, **Figure** **2**A,B). STEM analysis revealed an intriguing organization for all self‐assemblies, with phosphate salt concentrated within the core aiding in stabilizing the siRNA, while ultrasmall AuNC particles were uniformly distributed mainly on the periphery, as depicted in Figure 2C,D and Figure S6, Supporting Information, for AuSGR_2_ complexed with siRNA_TRF2_. Energy‐dispersive X‐ray spectroscopy measurements confirmed the presence of gold in each self‐assembly and the phosphate in the core of the assembly (Figure S7, Supporting Information), validating the relatively good monodispersity of these particles and the robustness of the assembly method. DLS data, reported in Table 1 and in Figure 2E, suggested a larger hydrodynamic diameter than the size obtained by microscopy, with an average size ranging between 122 and 220 nm in both water and PBS buffer. A quite broad distribution is observed for the assembly obtained with AuCR_9_ nanoclusters suggesting that the number of arginine may impact the size and stability of the complex with siRNA.

To confirm the size and morphology of such assemblies and to gather information on their structure in solution, we conducted small‐angle X‐ray scattering (SAXS) measurements on AuSGR_2_ complexed with siRNA_TRF2_. The significant scattering from both siRNA and gold allowed us to obtain SAXS profiles at high resolution. The pair‐wise distance distribution, *P*(*r*), obtained from the Fourier transformation of the SAXS profile, depicted in Figure 2F, confirmed an average size of 150 nm and suggested a nonspherical shape. The smooth decrease to 0 of *P*(*r*) also suggested an important degree of disorder that could be attributed to the flexibility of the siRNA_TRF2_ chains.

### 2.3 AuNCs Form Stable Self‐Assembled Complex with siRNA

When designing and developing a siRNA delivery system, it is crucial to consider factors such as binding affinity, siRNA stability, and surface charge as the most important determinants. The complexation of AuNCs with siRNA was analyzed by agarose gel electrophoresis (Figure 2G). The migration of siRNA in the gel was prevented in the presence of AuNCs indicating that the binding between the AuNCs and siRNA was strong enough to withstand dissociation. Optimization of the weight ratio of AuNCs to siRNA_TRF2_ to generate stable self‐assembled species indicated a decrease in this ratio as the positive surface of the AuNCs increased, with weight ratios of 8:1, 5:1, and 3:1 being utilized for AuSGR_2_, AuCR_3_, and AuCR_9_, respectively (Figure 2G). This behavior was expected, considering the number of arginines per AuNC necessary for electrostatic interaction with the siRNA (Table 1 and Figure S4, Supporting Information). These results seem to agree with calculation obtained through molecular dynamic simulations between atomically precise gold nanoclusters (Au_38_, Au_102_) and siRNA showing the strong influence of size and charge on their effective binding process with siRNA.^[^ ^32^ ^]^

It is well known that naked siRNA is susceptible to degradation when exposed to body fluids. We further analyzed the potency of self‐assembled AuNCs complexed with siRNA to prevent its degradation by RNase A using gel electrophoresis. As expected, naked RNA was subject to degradation by 1 μg mL^−1^ RNase A (Figure 2H well 4). Interestingly, RNA degradation with RNase A up to 10 μg mL^−1^ was prevented by its complexation with AuNCs (Figure 2H wells 5 to 9). Note that the concentrations of RNAse A being tested exceed the reported bloodstream concentration, which corresponds to 0.1 μg mL^−1^.^[^ ^33^ ^]^

Altogether, our results indicate that AuNCs are able to form complexes with siRNA and to efficiently protect RNA from RNase A degradation. Optimization of the AuNC‐siRNA weight ratio was found to be crucial for achieving stable and efficient formation of the assemblies.

### 2.4 Self‐Assembled AuNCs‐siRNA Showed Improved Optical Properties

The negatively charged (AuSG) and positively charged (AuSGR2, AuCR3, AuCR9) gold nanoclusters exhibit a typical absorbance profile in aqueous solution, characterized by strong absorption in the UV region that gradually decreases at longer wavelengths (Figure S8A, Supporting Information). This absorption profile remains unchanged over the course of one month, as demonstrated previously for AuSG and AuSGR_2_, and also observed for AuCR_3_ and AuCR_9_ (Figure S9, Supporting Information). Photoluminescence (PL) spectra reveal a broad emission with a peak at 610 nm for AuSG, indicating predominantly energy transfer from the surface of the AuNCs.^[^ ^34^, ^35^ ^]^ Using arginine‐rich peptides instead of glutathione led to a bathochromic shift of the PL emission from 610 to 660, 685, and 670 nm for AuSGR_2_, AuCR_3_, and AuCR_9_, respectively (Figure S8B,C, Supporting Information). This shift could be attributed to the modification of the density of ligand stabilizing the gold core that involves the interaction of the amine groups of arginine via intra‐ and interligands, as well as with the gold surface.^[^ ^30^, ^36^ ^]^ We estimated PL quantum yield (PLQY) from 5.0% for AuSG to 5.2%, 8.1%, and 6.2% for AuSGR_2_, AuCR_3_, and AuCR_9_, respectively. We hypothesize that PLQY increases with the presence of the arginine might facilitate a more efficient energy transfer between gold atoms and the ligand (ligand–metal charge transfer (LMCT), ligand–metal–metal charge transfer (LMMCT))^[^ ^14^, ^34^, ^37^ ^]^ and to the increase in ligand density on the gold surface of the AuNCs.^[^ ^30^, ^36^, ^38^ ^]^

**Figure 3:**
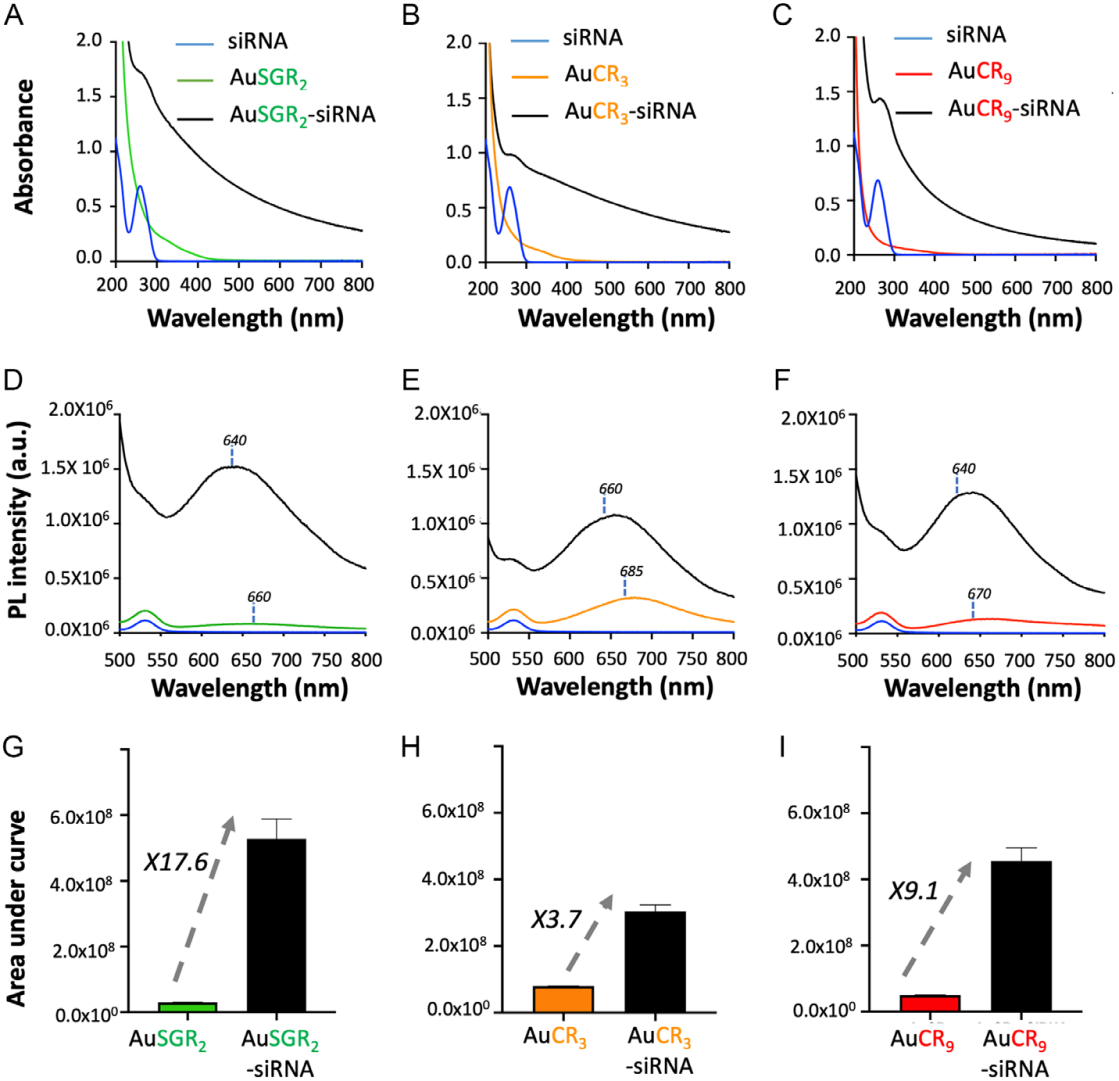
Optical properties of AuNCs complexed with siRNA. Absorbance spectra of siRNA, AuNCs, and self‐assembled AuNCs‐siRNA in RNAse‐free water using A) AuSGR_2_, B) AuCR_3_, and C) AuCR_9_. Photoluminescence (PL) spectra of siRNA, AuNCs, and self‐assembled AuNCs‐siRNA in RNAse‐free water using D) AuSGR_2_, E) AuCR_3_, and F) AuCR_9_ with ƛ_exc._ 450 nm. PL enhancement after self‐assembly determined after PL measurement at the same absorbance 0.1 (ƛ_exc._ 450 nm) in RNase‐free water using G) AuSGR_2_, H) AuCR_3_, and I) AuCR_9_ (*n* = 3).

When siRNA_TRF2_ is added to positively charged AuNCs, self‐assemblies are formed within the first minute, resulting in an overall increase in the absorbance spectra of AuNCs due to the scattering of the self‐assembled structures (**Figure** **3**A–C). The presence of siRNA is confirmed in the assemblies formed with the three types of AuNCs, indicated by a shoulder below 280 nm with a shift from 260 to 275 nm after assembly. We hypothesize that this blueshift is attributed to local distortion resulting from the electrostatic interaction between the positively charged AuNCs and the negatively charged siRNA. We monitored the evolution of the absorption band of siRNA at 260 nm over time for AuSGR_2_ complexed with siRNA_TRF2_ and AuCR_3_ complexed with siRNA_TRF2_ (Figure S10, Supporting Information). The shift of the absorbance band to 275 nm occurs within the first 30 min, then returns to 260 nm at 4 h, accompanied by a decrease in absorbance, indicating a possible disassembly and/or structural rearrangement of these structures.

There is a significant enhancement of the PL signal of the AuNCs when they form self‐assemblies with siRNA_TRF2_ (Figure 3D,F). The PL enhancement at shorter wavelengths confirms the formation of larger structures leading to strong scattering effects. The 20 nm blueshift of the maximum PL emission, which is typically observed in self‐assembled AuNCs, further supports this observation.^[^ ^22^, ^39^ ^]^ We determined the PL enhancement in diluted solutions for each self‐assembled AuNCs‐siRNA_TRF2_ as a function of the nature of the ligand stabilizing the AuNCs (Figure 3G–I) and repeated these measurements in three parallel experiments. Results showed a 3.8‐fold increase with AuCR_3_, a 17.6‐fold increase for AuSGR_2_, and a 9.1‐fold increase for AuCR_9_, attributed to the aggregation‐induced emission (AIE) effect,^[^ ^23^, ^40^, ^41^ ^]^ which could be explained by the stronger electrostatic interaction between AuNCs and siRNA due to the higher positive charge provided by the number of arginines. Therefore, the near distance between gold nanoclusters and the reduction of motion will favor energy transfer such as ligand‐to‐metal charge transfer (LMCT or LMMCT) leading to the boost of the PL in their assembly form.^[^ ^42^ ^]^ We hypothesize that the strongest AIE effect for the self‐assembly with AuSGR_2_ compared to AuCR_9_ or AuCR_3_ could be due to the efficient interparticle energy transfer from the rich electron donor of SGR_2_ due to the presence of glutathione^[^ ^43^ ^]^ compare to CR_*x*_ (*x* = 3, 9) ligand stabilizing the gold core of the AuNCs.^[^ ^22^ ^]^

### 2.5 AuNCs Mediate Efficient Cellular Uptake of siRNATRF2 That Downregulate TRF2 in Lung Cancer Cells

Prior to evaluating cellular uptake, the potential toxicity of AuSGR_2_, AuCR_3_, and AuCR_9_ was determined in a human lung adenocarcinoma cell line named A549 using PrestoBlue assays. It has been demonstrated that after IR exposure, A549 cell line shows a gradual dose‐dependent increase in radiation resistance and is therefore a pertinent model in the context of our study.^[^ ^44^ ^]^ As shown in Figure S11, Supporting Information, we observed that free AuNCs or complexed with siRNA did not elicit significant toxicity up to AuNC concentration of 100 μg mL^−1^, with the cell viability remaining above 70%. The cytotoxicity level was found also very low using another lung cancer cell line H358 in the same conditions indicating the possible use of such nanosystems to treat different types of lung cancer cells (Figure S11, Supporting Information). Surprisingly, we observed a decrease of cell viability in human fibroblast (MC5‐SV2) depending on the charge of AuNCs at concentrations exceeding 25 μg mL^−1^ that may be explained by the highly positive charge of nanocluster which can disrupt the membrane integrity of cells.

**Figure 4:**
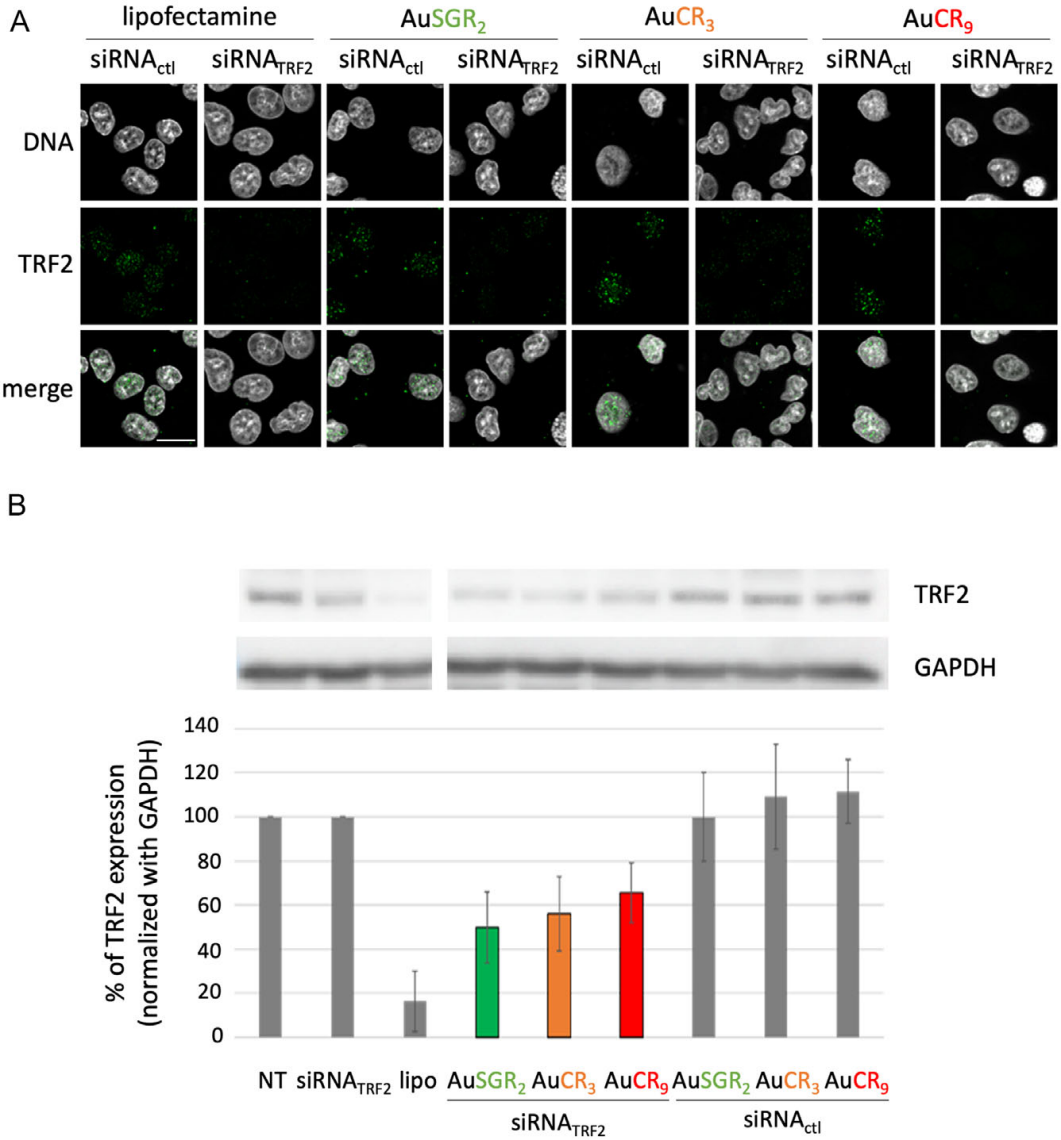
Efficient TRF2 downregulation in A549 cells line transfected with AuNCs‐siRNA_TRF2_. A) Immunofluorescence detection of TRF2 using fluorescence microscopy in cells transfected with the different self‐assembled AuNCs‐siRNA targeting TRF_2_ or a gene control. Lipofectamine combined with siRNA_TRF2_ was used as a positive control of TRF_2_ knockdown. DNA was stained with Hoechst. Scale bar corresponds to 10 μm. B) Western blot analysis and relative quantification of in vitro TRF_2_ expression in cells transfected or not (NT: nontreated) with the different self‐assembled AuNCs‐siRNA targeting TRF_2_ or a control gene nonexpressed in human cells (ctl). Lipofectamine (lipo) was used as a positive control of TRF_2_ knockdown. GAPDH was used as a loading control. Relative quantification of TRF_2_ expression is normalized with GAPDH and expressed as a % of TRF2 expression level in NT cells.

To determine whether cellular uptake of AuNCs‐siRNA translates into effective neutralization of endogenous TRF2 mRNA and protein expression, we next assessed the knockdown efficacies of TRF2 in A549 cells in comparison with control sequence targeting a gene that is not expressed in human (siRNA_ctrl_) by immunofluorescence (**Figure** **4**A) and by western blot 72 h post‐transfection (Figure 4B) as reported in the literature.^[^ ^4^ ^]^ All siRNA were designed with modifications as 2′‐methoxy modifications in both strands and phosphorothioate at 3′ extremities to improve chemical stability, to reduce both immunological effects and off‐target effects.^[^ ^45^ ^]^ First, we demonstrated the ability of the various positively charged AuNCs complexed with siRNA_TRF2_ to deliver siRNA_TRF2_ to the cellular cytoplasm using immunofluorescence combined with fluorescence microscopy (Figure S12, Supporting Information). This delivery resulted in the selective and efficient knockdown of TRF2, as illustrated in Figure 4A, where a weak TRF2 signal was detected 72 h after cellular transfection. A similar experiment was performed with AuSGR_2_ complexed with siRNA_TRF2_ in H358 cell lines (Figure S13, Supporting Information), confirming the downregulation of TRF2 in another lung cancer cell line.

Western blot analysis was performed to detect TRF2 and quantify TRF2 expression level (Figure 4B). The AuNCs‐siRNA (300 pmol of siRNA) that exhibited the best downregulation of TRF2 expression was AuSGR_2_‐siRNA_TRF2_ with a 50% TRF2 downregulation compared to the nontreated control. AuCR_3_‐siRNA_TRF2_ and AuCR_9_‐siRNA_TRF2_ induced around 40% of TRF2 knockdown. In contrast, the complex AuNC‐siRNA_ctl_ did not significantly inhibit TRF2 expression. Free siRNA_TRF2_ showed no TRF2 inhibition. The Lipofectamine 2000 transfection reagent‐mediated siRNA delivery validates the functionality of siRNA_TRF2_ designed with more than 80% of TRF2 expression decrease. However, Lipofectamine 2000 is known to induce significant toxicity to various cell types^[^ ^46^ ^]^ and might limit their translation for in vivo studies compared to AuNCs. We need to consider that the TRF2 knockdown was evaluated for the total cell population that have been exposed to AuNC‐siRNA. Some cells may not be transfected with the nanosystem or may not incorporate the same quantity of AuNC‐siRNA.

Based on the optical properties, cytotoxicity experiments, and downregulation efficiency of AuNCs‐siRNA_TRF2_, we selected AuSGR_2_‐siRNA_TRF2_ as the candidate for further cellular studies.

**Figure 5:**
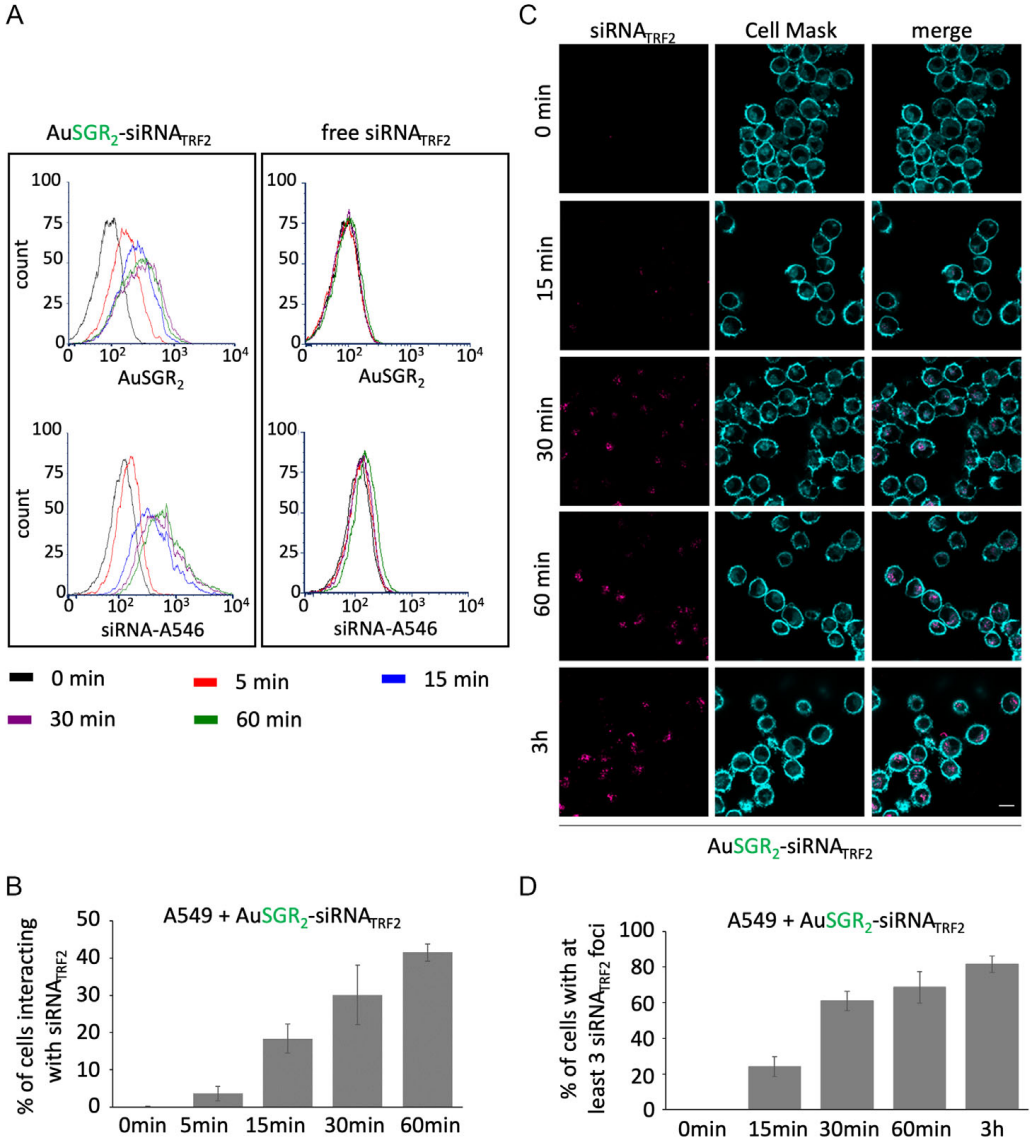
Kinetic of cellular interaction and efficient cellular uptake of AuSGR_2_ complexed with siRNA in A549 cells. Cells were incubated or not for different time periods with A546‐labeled siRNA_TRF2_ free or complexed with AuSGR_2_, A) analyzed by flow cytometry and B) quantified for positive cells interacting with siRNA_TRF2_. (*n* = 2). C) Cells were incubated with Cy5‐labeled siRNA_TRF2_ complexed with AuSGR_2_ at different times and followed by fluorescence microscopy for quantification (*n* = 3). D) CellMask was used to stain the cells (scale bar: 10 μm).

To gain insight into the kinetics of cellular interaction of AuSGR_2_‐siRNA, flow cytometry analysis was performed to measure the cellular fluorescence intensity of AuSGR_2_ and siRNA_TRF2_ labeled with Alexa‐546 dye (siRNA‐A546) after 0, 15, and 30 min and 1 h of incubation with A549 cells (**Figure** **5**A,B) and H358 cells (Figure S14, Supporting Information). Our results clearly show an increase in both siRNA_TRF2_‐A546 dye and AuSGR_2_ signals in the two different lung cancer cell lines tested, in a time‐dependent manner (Figure 5B,C and Figure S14, Supporting Information). siRNA_TRF2_ interacted with 40 to 60% of the cells after 1 h of incubation with AuSGR_2_‐siRNA. No significant fluorescent signal was detected in cells incubated with free siRNA_TRF2_.

Next, we analyzed the kinetics of AuSGR_2_‐siRNA_TRF2_ cellular uptake in A549 cells using fluorescence microscopy (Figure 5C). The results in Figure 5D indicated that AuSGR_2_ could deliver labeled Cy5‐siRNA_TRF2_ to the cellular cytoplasm in up to 20% of cells after only 15 min of incubation, and in almost 80% of cells after 1 h of incubation, suggesting widespread diffusion of the gold nanoclusters.

Our results demonstrate that AuNCs were able to protect siRNA from degradation and facilitate their cellular uptake and delivery for effective siRNA‐mediated gene silencing in a time manner dependent without significant cellular toxicity.

### 2.6 AuSGR2‐siRNATRF2 Increase Radiosensitivity in Lung Cancer Cells

**Figure 6:**
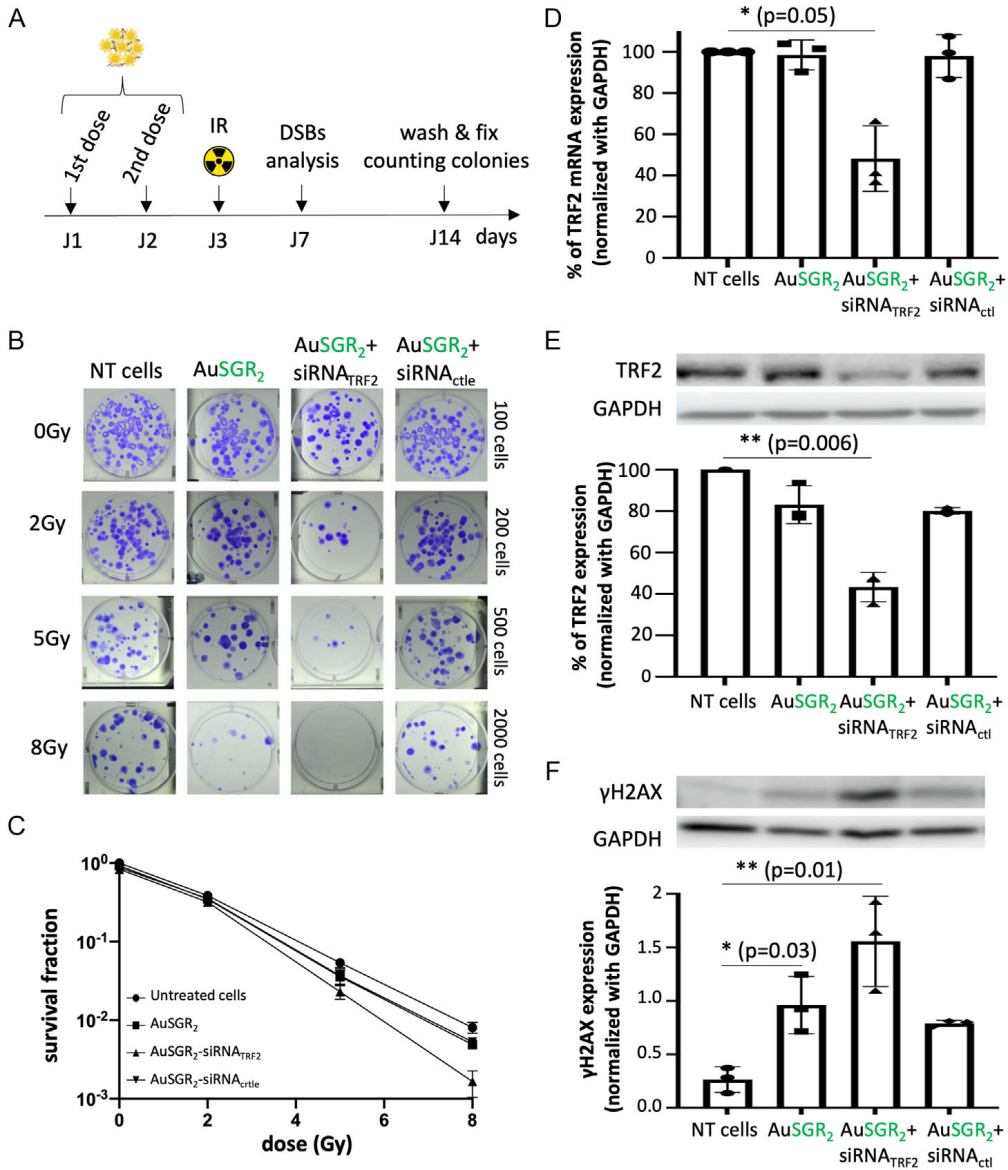
Self‐assembled AuSGR_2_‐siRNA_TRF2_ treatment sensitizes A549 cells to IR. A) Clonogenic assay. Cells were treated with 2 doses of AuSGR_2_‐siRNA at 1 day interval. 24 h after the second dose, the cells were seeded at different cell concentrations and then irradiated under different doses (0, 2, 5, and 8 Gy). The siRNA_ctrl_ was used as a negative control that has no target in the cells. The formation of DNA DSBs was analyzed 96 h after irradiation. The cells were cultured for 2 weeks, and the surviving colonies were counted. B) Representative clonogenic survival images of cells at the different conditions tested were treated 2 weeks after irradiation. C) The effect of combinational treatment AuNCs and siRNA_TRF2_ on the cell colony formation of irradiated or nontreated cells was analyzed using a survival fraction curve. The impact on AuSGR_2_ complexed or not with siRNA_TRF2_ on D) TRF2 mRNA level, on E) TRF2 protein expression, and on F) γH2AX protein expression in cells 96 h after irradiation at 8 Gy was analyzed by qPCR and western blot. TRF2 and γH2AX expressions were quantified by normalizing with the loading control GAPDH. The results are represented as the mean of three independent experiments, with each performed in triplicate (*n* = 3). The error bars signify standard deviation (SD).

For the following experiments, we selected the best AuNC‐siRNA candidate based on its ability to knock down TRF2 expression, its strongest photoluminescence, and its lower hydrodynamic diameter. In order to examine whether AuSGR_2_ alone or in combination with siRNA_TRF2_ enhance the potency of irradiation, A549 cells were treated with nontoxic concentrations of AuNPs or/and siRNA_TRF2_ and received 0, 2, 5, and 8 Gy radiation doses (**Figure** **6**A). The colony‐forming capability of the samples was then determined. Cells were transfected with AuSGR_2_ at 8 μg mL^−1^ and 300 pmol of siRNA twice at a weight ratio (8:1), in 24 h time intervals, starting from 24 h after seeding. The surviving fractions (SF) were calculated by normalizing the plating efficiency of irradiated cells to the corresponding nonirradiated and nontreated ones (0 Gy and in the absence of AuSGR_2_) (Figure 6B). The analysis of radiobiological parameters from the survival curves presented in **Table** **2** reveals that, at a low dose (2 Gy), AuSGR_2_ alone had negligible impact on cell survival. However, at 5 Gy, AuSGR_2_ demonstrates a 1.5‐fold decrease in cell survival compared to untreated cells. The survival fractions of cells treated with AuSGR_2_ complexed or not with siRNA_ctrl_ were significantly lower than those of untreated cells across all the tested radiation doses with a *p* value = 0.02 (Figure 6C). This result confirms the radiosensitization potential of AuNCs as it was demonstrated in other studies.^[^ ^47^ ^]^ Compared with cells treated with AuSGR_2_, the survival fractions of cells treated with AuSGR_2_‐siRNA_TRF2_ were consistently lower at each tested radiation dose with a *p* value < 0.01 (Figure 6C). When combined with siRNA_TRF2_, AuSGR_2_ induces a 2.3‐fold decrease in survival fraction at 5 Gy (Table 2). These results are in adequation with the study that demonstrated that downregulation of TRF2 in A549 cells was associated with increased radiosensitivity.^[^ ^5^ ^]^ These data suggest a synergistic effect between the nanoparticle and the siRNA_TRF2_ to favor radiosensitivity in A549 cells.

**Table 2:** Radiobiogical parameters determined by survival curves. The alpha and beta values for each experimental condition (cells treated or not with AuSGR_2_‐siRNA_TRF2_ or AuSGR_2_ or AuSGR_2_‐siRNA_ctle_ were calculated from the radiation survival curves using the linear quadratic model of fitting (SF = exp − (*αD* − *βD*2)) using least mean squares minimization). Statistical errors on fit values were calculated with 95% confidence intervals. SF2 and SF5 indicate the surviving fraction at 2 and 5 Gy respectively. *R* ^2^ represents the proportion of the variation in the dependent variable that is predictable from the independent variable(s) of the survival curve.

	*α* (Gy^−1^)	*β* (Gy^−1^)	*α*/*β*	SF2	SF5	*R* ^2^
No treatment	0.4050	0.0350	11.55	0.380 ± 0.023	0.054 ± 0.007	1
AuSGR_2_‐siRNA_TRF2_	0.4387	0.0448	9.775	0.347 ± 0.023	0.035 ± 0.007	0.9901
AuSGR_2_	0.4514	0.0604	7.466	0.254 ± 0.080	0.023 ± 0.004	0.9404
AuSGR_2_‐siRNA_ctl_	0.4281	0.0459	9.32	0.353 ± 0.016	0.036 ± 0.008	0.9701

Cell morphology observations and apoptosis assays were conducted both before and 96 h after irradiation of A549 cells, which were incubated with either AuSGR_2_ alone or AuSGR_2_ complexed with siRNA_TRF2_, as well as a control siRNA (Figure S15A,B, Supporting Information). Our results indicate that treatment with AuSGR_2_, either alone or complexed with siRNA_TRF2_, impacts cell morphology following irradiation. However, no apoptosis was induced, suggesting that a different cellular mechanism is responsible for the arrest in cell proliferation observed in Figure 6.

In parallel, to verify the ability of AuSGR_2_ to deliver siRNA_TRF2_ in the tested conditions tested, relative expression levels of TRF2 were evaluated by RT‐qpCR (Figure 6D) and by western blot (Figure 6E) analyses 48 h after the first AuSGR_2_‐siRNA incubation with cells. We confirmed the efficient 50% downregulation of TRF2 expression in cells incubated with AuSGR_2_‐siRNA_TRF2_ by both techniques.

Previous studies have demonstrated that TRF2 protects the telomeres from end‐to‐end fusion and plays important roles in DNA damage response induced by radiation by serving as a survival factor in telomere maintenance.^[^ ^48^, ^49^ ^]^ It was suggested that TRF2 regulates radiosensitization through telomere length and DNA damage response. TRF2 is required to prevent activation of the DNA damage response pathway at chromosome ends. Therefore, TRF2‐depleted telomeres elicit DNA damage response. If AuSGR_2_ and siRNA_TRF2_ together augment the cellular damage caused by IR, the amount of DNA double‐strand breaks (DSBs) should also increase due to the combined treatment. The visualization of phosphorylated histone γ‐H2AX as a biomarker for DSBs in response to IR was used to quantify radiation‐induced DSBs in cells with a low TRF2 expression. DNA damage response was detected with an anti‐γ‐H2AX using western blot analysis 96 h after the irradiation (Figure 6F). Indeed, it has been demonstrated that in TRF_2_ partially knockdown cells, the level of γ‐H2AX was similar to control cells within the first 48 h after the irradiation, whereas when incubation time extended to 72 h, γ‐H2AX levels showed a significant increase.^[^ ^4^ ^]^ Clear evidence of DNA damage was observed in irradiated cells treated with AuSGR_2_‐siRNA_TRF2_ compared to untreated cells. Additionally, the phosphorylated form of γ‐H2AX was detected in irradiated cells treated with AuSGR_2_, independently of the presence of siRNA, although to a lesser extent than in cells treated with AuSGR_2_‐siRNA_TRF2_ (Figure 6F). We hypothesize that the radiosensitizer effect of AuSGR_2_ may favor the formation of DNA DSBs in irradiated cells. The low detectable level of DSBs in unirradiated cells was likely due to the genomic instability and replication stress common to cancer cells.^[^ ^50^, ^51^ ^]^

No significant apoptosis was detected in irradiated cells treated with AuSGR_2_ complexed with siRNA_TRF2_. TRF2 is essential for telomere protection, and previous studies have shown that partially deprotected telomeres activate a DNA damage response (DDR), primarily inducing replicative senescence^[^ ^52^ ^]^ rather than apoptosis. Unlike apoptotic cells, senescent cells remain viable and undergo permanent cell cycle arrest. Additionally, Orun et al.^[^ ^4^ ^]^ demonstrated that partial TRF2 knockdown in immortalized human mesenchymal stem cells exposed to radiation increases senescent cell counts and alters cell morphology.

Our study demonstrates that the DNA damage resulting from radiation was increased by TRF2 low expression and the presence of AuNCs in A549 cells. Taken together, our results indicate that TRF2 downregulation as well as the radiosensitizer AuNCs enhanced the response of lung cancer cells to radiation by reducing clonogenic cell survival and by decreasing DSB repair efficiency.

## 3 Conclusion

We designed, synthesized, and self‐assembled stable gold nanoclusters complexed with siRNA. They are capable of entering cells and delivering biologically active siRNA in the cytoplasm that target gene expression and reduce down to 50% TRF2 expression. We demonstrated that the number of arginine covalently bound to AuNCs complexed with siRNA influences the stability and the photoluminescence intensity of the assembly. X‐ray exposure of lung cancer cells demonstrates a 2.3‐fold improvement of cell death in the presence of the self‐assembled nanosystems with a synergistic effect between the knockdown of TRF2 and the radiosensitizing properties of AuNCs. We conclude that TRF2 downregulation, along with AuSGR_2_ presence in irradiated cells, enhances the radiosensitivity of tumor cells by decreasing DSB repair efficiency and reducing cell proliferation, likely due to senescence activation in our study.

While such results are promising and gold nanoclusters have been widely considered as “safe theranostic agents,”^[^ ^25^, ^53^, ^54^ ^]^ there are still some aspects that need a deeper investigation to fully envision a potential clinical translation. First, the transfection efficacy should reach more than 80% and the fate of the gold nanoclusters in cells (exocytosis, biomineralization) and their cytotoxicity need to be evaluated in the long term. Secondly, for future in vivo studies, colloidal stability preventing degradation of the siRNA by RNase and recognition by the immune system as well as the biodistribution of such assemblies should be investigated. Third, a key aspect to reduce toxicity will be to add targeting properties to such assemblies without affecting the capacity to form assembly and deliver siRNA efficiently. With their diagnostically useful optical features and biological inertness, AuNC‐siRNA_TRF2_ appear as a promising tool to enhance radiosensitivity in the RT of cancer cells activating the telomeric protective response.

## 4 Experimental Section

4.1

4.1.1

##### Materials

All chemical products were bought from Sigma–Aldrich (France) including the tripeptide Glutathione SG. The peptides SGR_2_ (Glu‐Cys‐Gly‐Arg‐Arg; 619 g mol^−1^), CR_3_ (Cys‐Arg‐Arg‐Arg; 589 g mol^−1^), and CR_9_ (Cys‐Arg‐Arg‐Arg‐Arg‐Arg‐Arg‐Arg‐Arg‐Arg; 1527 g mol^−1^) were prepared in GenScript (Netherlands) with high purity (95%). Syntheses were performed using deionized water.

##### Synthesis of AuNCs

AuSG and AuSGR_2_ (also named Au_22_SG_18_ and AuSG‐2Arg, respectively^[^ ^30^ ^]^) were prepared as described previously.^[^ ^30^ ^]^

Ultrasmall gold nanoclusters AuCR_3_ stabilized by 25% of SG and 75% of CR_3_ were synthesized using a molar ratio Au: Ligand: NaBH_4_ = 1: 2: 0.0125. Briefly, CR_3_ and SG were mixed in 3 mL of water and 96 μL of HAuCl_4_ in solution (20 mM) was added under rapid stirring at 500 rpm. pH was then adjusted at 9 with NaOH (1 m) and freshly prepared reducing agent NaBH_4_ (1 mM) was added dropwise and kept stirred for 30 min. pH was lowered down to 2 by adding HCl (1 m) and the solution was kept under stirring for another 4 h at 350 rpm. AuCR_3_ was precipitated in a mixture of water/ethanol (30/70) and washed 3 times with a 3 kDa Amicon filter to remove the unreactive products. The solution of AuNCs was then lyophilized for storage at room temperature. AuCR_9_ was prepared with a similar protocol as AuCR_3_.

##### Preparation of Self‐Assembled AuNCs‐siRNA

The positively charged AuNCs were mixed with siRNA solution in RNase‐free water and shaken on a bench‐top shaker for 30 min to complete the binding of siRNA with AuNCs via electrostatic interaction. 5 μg of siRNA (corresponding to 300 picomol) was added to AuNCs at different optimized weight ratios: 8:1 ratio for AuSGR_2_:siRNA, 5:1 ratio for AuCR_3_:siRNA, and 3:1 ratio for AuCR_9_:siRNA. The prepared samples were abbreviated as AuNCs‐siRNA in this study. We used chemically modified nucleotides synthesized by Eurogentec.^[^ ^45^ ^]^ Double‐stranded TRF2 small interfering RNA (siRNA) sequences were designed as follows: 5′‐CCU UCU UUA GUG GUU UGC UUA UTT‐3′ (sense) and 5′‐AUA AGC AAA CCA CUA AAG AAG GTT‐3′ (antisense). The sequences of control were designed as 5′‐GCUUGAAGUCUUUAAUUATT‐3′ (sense) and 5′‐TAAUUAAAGACUUCAAGCTT‐3′ (antisense). Double‐stranded siRNA used contains two 2′‐methoxy modifications at the positions 5 and 12 for siRNA_TRF2_ sequence and at the positions 4 and 12 for siRNA_ctrle_ sequence in both strands to improve siRNA stability and siRNA binding affinity with AuNPs, as well as to reduce the innate immune response. The two last phosphodiester linkage at 3′ ends were replaced by two phosphorothioates to protect siRNA from 3′‐exonucleases in vitro and in vivo.^[^ ^55^ ^]^ Alexa546‐siRNA_TRF2_ and Cy5‐siRNA_TRF2_ were synthesized and purified by Eurogentec.

##### Physicochemical Characterizations of AuNC‐siRNA Conjugates

Morphologies of the self‐assembled AuNC‐siRNA samples were obtained on Scanning transmission electron microscopy (STEM) using a FEG JEOL 2100F system at 200 kV. EDX measurements were performed to detect gold and phosphate elements within the self‐assemblies. Sizes of the metal cores of AuNCs were determined by HRTEM JEOL2010 using a monochromated microscope working at 200 kV. Prior to imaging, the AuNCs and AuNC‐siRNA were dispersed on copper grids covered with a carbon film. Particle size in each condition was estimated on at least 100 particles using FIJI software.

SAXS data were collected at the SWING beamline at SOLEIL synchrotron in France.^[^ ^56^ ^]^ A highly concentrated solution was prepared by slowly mixing 6.1 μL of siRNA (100 μM) with 18.6 μL of AuSGR_2_ (2 mg mL^−1^) in 174.6 μL of RNase‐free H_2_O. After 30 min at room temperature, additional dilutions were made using free RNase water (1/100, 1/50, 1/10, 1/5). Subsequently, 40 μL of the samples were directly injected into a 1.5 mm diameter and 10 μm thick‐walled quartz capillary using a robotic arm equipped with electronic pipettes. Buffer measurements were conducted before and after each sample injection. Samples were measured using the in‐vacuum EigerX 4m detector with a distance of 6 m and a photon energy of 12 keV, enabling a *q* range of 1.4 × 10^−3^ to 0.185 Å^−1^ (with *q* = 4πsinθ/λ).

Raw image processing, including masking and azimuthal averaging, was performed using the FOXTROT program.^[^ ^57^ ^]^ Buffer subtraction, curve averaging, and SAXS profile analyses were done with the ATSAS 3.2.1 software package,^[^ ^58^ ^]^ which includes AUTORG for calculating the radius of gyration (*R*_g_) and GNOM^[^ ^59^ ^]^ for the calculation of pair‐wise distance distribution, *P*(*r*). Only the curve measured at 1/10 dilution, having enough signal‐to‐noise and not displaying interparticle interactions, is presented here.

The hydrodynamic diameter and the zeta potential of the prepared nanomaterials dispersed in water and phosphate buffer (PBS 10 mM, pH 7.4) were measured in triplicate on a Zetasizer instrument from Malvern.

NMR‐DOSY experiments were carried out at 298 K on a Bruker AVANCE III 400 MHz spectrometer. The sample concentration was about 2 mM in D_2_O. The standard “ledbpg2s” Bruker sequence was used with linear gradient stepped between 2 and 98%. Sixty‐four scans were recorded for each gradient step. Diffusion coefficient *D* was obtained by processing the data with the maximum entropy algorithm from Dynamics Center, a Bruker's NMR software, by using the peak area. An average value of D was used for the hydrodynamic diameter (*d*) calculation according to the Stokes–Einstein equation which assumes that molecules are spherical

(1) $$d=k_{B}T/3D\pi \eta$$

where *k*_B_ is the Boltzmann constant, *T* is the temperature, and *η* is the viscosity of the solvent (*η*_D2O_ *= *1.232 10^−3^ Pa.s at 298 K).

Electrophoretic mobility shift assay in 4% of agarose gel containing Tris‐glycine buffer at pH 7 was performed to evaluate the complexation between AuNCs and siRNA as well as the protection of siRNA against RNase degradation. Free siRNA (1 μg) and AuNC‐siRNA complex were incubated or not at 37 °C with RNase A at different concentrations (1, 5, and 10 μg mL^−1^) for 5 min. Samples were then incubated with RNase inhibitors (RNAsecure) at 60 °C for 15 min to inactivate RNase. After adding glycerol, each sample was loaded into the agarose gel and the voltage was applied at 100 V for 30 min. Fluorescent AuNCs were detected at 750 nm. In a second time, the resulting gel was stained with SYBR‐gold (Invitrogen, S11414) or GelRed (Biotium, 41003) and siRNA was visualized on a Gel Doc imaging system (Bio‐Rad).

##### Optical Characterizations of AuNC‐siRNA Conjugates

All spectroscopic measurements were done in 1 cm quartz cuvettes from Hellma GmbH at room temperature using air‐saturated solutions. The absorption spectra of diluted AuNCs and AuNC‐siRNA samples in water and in PBS buffer (10 mM) were recorded on Perkin Elmer Lambda 1050 UV‐vis spectrophotometer between 190 and 800 nm. Steady‐state photoluminescence spectra were measured from 500 to 800 nm on a Fluoromax (Perkin Elmer) spectrofluorometer. Determination of the PL emission enhancement was estimated using the area under each curve on a Graphpad (Prisms) software.

With the aim to estimate the quantum yields (QY) of the Au NCs, fluorescence spectra of the reference DCM dye (4‐(dicyanomethylene)‐2‐methyl‐6‐(4dimethylaminostyryl)‐4H‐pyran) and Au NCs were recorded in methanol and in water respectively, between 430 and 800 nm, (*λ*_exc_ = 410 nm). QY were calculated using the following formula.

(2) $$\text{QY}={\text{QY}}_{\text{ref}}|\bar{\frac{\eta^{2}}{\eta_{\text{ref}}^{2}}\frac{I}{I_{\text{ref}}}\frac{A_{\text{ref}}}{A}}$$

where, QY_ref_ = quantum yield of the reference DCM dye (QY = 44%, *λ*_em_ = 615 nm, in methanol)

*η* = refractive index of H_2_O (1.333)

*η*_ref_ = refractive index of MeOH (1.326)

*I* = integration of the emission spectra for the sample

*I*_ref_ = integration of the emission spectra for the DCM dye

*A* = optical density (0.05 OD at *λ*_exc_ = 410 nm for the sample and the reference dye).

##### Cell Culture

A549 cell line (lung adenocarcinoma epithelial cells; ATCC CCL‐185) and H358 cell line (human bronchioalveolar carcinoma) were maintained in RPMI culture medium (Gibco‐Life Technologies) supplemented with 10% inactivated fetal calf serum and kept at 37 °C in a humid atmosphere with 5% CO_2_. MRC5‐SV2 cell line (fibroblasts from the human lung) was maintained in MEM culture medium (Gibco‐Life Technologies) supplemented with 10% inactivated fetal calf serum, 1% NEAA, and 1% sodium pyruvate and kept at 37 °C in a humid atmosphere with 5% CO_2_.

##### Transfection

Cells were seeded in 6‐well plates at 1.10^5^ cells per well. When the cells reached 50% confluence, the culture medium was removed and replaced by 1 mL of OptiMEM culture medium (Gibco‐Life Technologies). In parallel, 300 pmol of siRNA_TRF2_ or siRNA_ctrle_ were mixed with AuNCs at different weight ratios (see below). Complexation of siRNAs with AuNCs and formation of self‐assembly were carried out in 200 μL of RNase‐free water at pH 7, for 30 min at room temperature. Lipofectamine RNAiMax (Thermo Fisher) reagent was used as a positive transfection control and mixed with siRNAs according to supplier protocols. Transfection mix was then added dropwise to each well and cells were incubated for 4 h at 37 °C. Finally, 2 mL of RPMI 10% FBS culture medium was added to each well, and the cells were incubated at 37 °C. After 24 h, a second dose of the transfection mix produced under the same conditions was added to the cells, which were then left for a further 24 h at 37 °C. Each condition was performed at least three times in independent manipulations.

##### Cytotoxicity Assays

Cell viability was measured using the PrestoBlue Cell Viability Reagent from Invitrogen, according to the manufacturer's instructions. Briefly, cells were seeded in 96‐well plates and incubated with different concentrations of AuNPs (from 25 to 100 mg mL^−1^) for 72 h. 10 μl of PrestoBlue solution was then added to each well for 1 h at 37 °C in a humidified, 5% CO_2_ atmosphere. The reducing environment within viable cells converts the nontoxic resazurin in the PrestoBlue reagent to an intensely red fluorescent dye. This change was detected by measuring absorbance at 570 nm using a CLARIOstar Plus microplate reader (BMG Labtech).

##### Flow Cytometry Analysis

A549 or H358 cells were seeded in 6‐well plates at a density of 200 000 cells/well. The next day, the culture medium was replaced by 300 pmol of A546‐labeled siRNA_TRF2_ free or complexed with AuSGR_2_ at a weight ratio of AuNCs to siRNA of 8:1 in 1 mL of OPTIMEM (Gibco‐Life Technologies). Cells were incubated during different time periods (0, 5, 15, 30, and 60 min) at 37 °C. The cells were washed in PBS 1X, trypsinized, and washed again in PBS 1X. The cells were then fixed in PFA 4% for 10 min at room temperature and washed 2 times in PBS 1X before being resuspended in PBS 1X for flow cytometry analysis. Fluorescence intensities of the cell population that interact with A546‐labeled siRNA_TRF2_ and AuSGR_2_ were measured respectively with *λ*_exc_ = 488 nm or 562 nm and with *λ*_em_ = 573 or 790 nm. The data were analyzed using FCS Express 4.

##### Fluorescence Microscopy

Twenty‐four hours before the experiment, 1.10^5^ cells were seeded in Nunc Lab‐Tek 4‐well coverslips (Thermo Fisher; 155383).

For immunofluorescence detection of TRF2, cells were transfected 2 times as mentioned below. Forty‐eight hours post‐transfection, cells were washed in PBS 1X and permeabilized with a permeabilization buffer (20 mM Tris‐HCl (pH = 8), 50 mM NaCl, 3 mM MgCl2, 300 mM Sucrose, 0.5% Triton X‐100) for 5 min. They were then fixed in 4% paraformaldehyde in PBS 1X for 15 min and washed 3 times in PBS 1X. To prevent nonspecific binding of antibodies, cells were incubated in a blocking buffer (SVF10% in PBS 1X) for 30 min at room temperature. Subsequently, cells were incubated with mouse polyclonal α‐TRF2 antibody from Novus Biological (1:200 dilution in blocking buffer) for 1 h at 37 °C. Cells were then washed 3 times for 5 min in PBS 1X‐triton 0.1% at room temperature and incubated with Alexa488‐labeled anti‐mouse IgG antibody from Cell Signaling (1:500 dilution in blocking buffer) for 1 h at 37 °C. After 3 washes for 5 min in PBS 1X‐triton 0.1%, DNA was stained with Hoechst 33258 at 5μM for 5 min at room temperature.

For fluorescence detection of Cy5‐labeled siRNA, cells were incubated with free siRNA or with AuNC‐siRNA conjugates at optimized RNA to AuNC weight ratios for 3 h at 37 °C. After three washes in PBS 1X, cells were incubated with CellMask Green (Thermo Fisher) diluted 1:1000 for 15 min at 37 °C in RPMI culture medium without phenol red (Gibco‐Life Technologies) for actin staining. Cells were then fixed with 4% paraformaldehyde (Sigma–Aldrich) diluted in PBS 1X for 10 min at 37 °C and washed three more times with PBS 1X. Cellular internalization of AuNC‐siRNAs complexes was visualized using a Zeiss LSM7 Live microscope (Dynascope) at the ×64 objective under immersion (oil). Fluorescence quantification was performed with ImageJ software. The same protocol was followed for the kinetics of AuSGR_2_‐siRNA cellular uptake at different incubation time periods of 5, 15, 30, 60, and 180 min.

##### Radiation of Cells

Cells were exposed to radiation in an X‐ray generator device (SARRP device from Xstrahl). The parameters applied to all groups were as follows: voltage 220 kV, current 13 mA, and a distance between the source and the sample of 35 cm. After irradiation, culture medium was changed immediately. RT was performed using the Grenoble MRI facility IRMaGE—SAXO platform.

##### CFU Assay

Cells were transfected with AuSGR2 complexed or not with siRNA_TRF2_ and siRNA_ctrle_, washed in PBS 1X after 48 h, trypsinized, and plated at appropriate dilution (between 50 and 2000 cells/well) in a 6‐well plate as three replicates. Each group was irradiated at the dose point of 0, 2, 5, and 8 Gy, respectively. After 14 days of incubation at 37 °C in 5% humidified CO_2_, the colonies were washed with PBS 1X, fixed, and stained with a mixture of 6% glutaraldehyde and 0.5% Crystal Violet for 30 min at room temperature. Visible colonies were counted after three washes in H_2_O. Those colonies containing ≥50 cells were scored as viable colonies. Survival curves for each group were demonstrated using GraphPad Prism 5.0 software. Radiobiological parameters, such as γ and β, were calculated according to the survival curves.

##### Western Blot Analysis of TRF2 and γH2AX

Cell lysis was performed in NP40 buffer (Tris 20 mM (pH = 7.5), NaCl 150 mM, EDTA 2 mM, NP40 1%) on ice and then cells were sonicated for 5 min (30 s ON, 30 s OFF) at 4 °C. Protein extracts were obtained after 1 min centrifugation at 14 000 rpm at 4 °C. Total protein extracts were quantified by spectrophotometry using a Bradford assay. Equal amounts (15 to 30 μg) of whole protein extracts were loaded and separated on 10% or 15% polyacrylamide gels. Primary antibodies against TRF2 (Novus, NB100‐56506) and γ‐H2AX (Abcam, ab11174) were used and diluted in PBS 1X, BSA 1%. Membranes were washed in PBS 1X Tween 0.1% and then incubated with secondary antibodies antimouse or antirabbit IgG linked with HRP. For loading controls, anti‐GAPDH antibodies (Santa Cruz, 365062) were used. Target protein signal was obtained using ECL (GE Healthcare) and revealed using ChemiDoc MP System (Bio‐Rad). Band intensities were quantified using Image‐J software.

##### RT‐qPCR Analysis of TRF2

The cells in different conditions were harvested and total RNA were isolated using the RNAeasy plus kit with DNase treatment from Qiagen following the manufacturer's instructions. 1 μg of RNA was reverse transcribed with an equal amount of oligonucleotide (dT)15 primer and oligonucleotide random hexamers using GoTaqTM probe 2‐step RT‐qPCR system from Promega according to the manufacturer's instructions. Controls without reverse transcriptase or RNA were performed. For quantification of TRF2 transcripts, real‐time PCR analysis was performed with the following conditions: one cycle of 95 °C for 2 min, 45 cycles of 95 °C for 15 s, 55 °C for 30 s, and 60 °C for 60 s. The sequences of forward primers and reverse primers are indicated in the **Table** **3**.

**Table 3:** Sequences of forward and reverse primers for TRF2 and GAPDH proteins.

Names of primers	Sequences
TRF2 forward	5′‐GCTGCCTGAACTTGAAACAGT‐3′
TRF2 reverse	5′‐CCGTTCTCAACCAACCCCTC‐3′
GAPDH forward	5′‐AGCCACATCGCTCAGACAC‐3′
GAPDH reverse	5′‐GCCCAATACGACCAAATCC‐3′

Q‐PCR was performed on a CFX Opus 384 real‐time PCR machine (Bio‐Rad) and analysis using the CFX Maestro software. Primer binding specificity was verified by performing their melting curves (Figure S16, Supporting Information).

##### Observation of Cells Morphologies

Cells treated or not with AuSG‐R2 and complexed or not with siRNATRF2 were seeded in 6‐well plates and observed 96 h after irradiation using a phase contrast microscope.

##### Analysis of Caspase‐3 Activity

The percentages of cells in apoptosis were determined using the FITC Active Caspase‐3 Apoptosis Kit (BD Pharmingen) followed by quantification of the fluorescent‐labeled antibody that specifically recognizes the active form of caspase‐3 in human cells using an Attune flow cytometer.

##### Statistical Analyses

Statistically significant differences were defined at a level of *p* < 0.05. * means *p* = 0.05; ** means *p* = 0.002, and *** means *p* < 0.001. For western blot analysis, Kruskal–Wallis tests were used to determine the statistical significance of the results. For the CFU assay, the statistical significance of mean differences in survival fractions curve between the control and experimental groups was tested using two‐way ANOVA multiple comparisons.

## Conflict of Interest

The authors declare no conflict of interest.

## Author Contributions

**Sean Moro**: formal analysis (lead); investigation (lead); writing—original draft (supporting). **Mohamed Omrani**: formal analysis (supporting); investigation (supporting). **Sule Erbek**: formal analysis (supporting). **Muriel Jourdan**: formal analysis (supporting); validation (supporting). **Catharina I. Vandekerckhove**: formal analysis (supporting); investigation (supporting). **Cyril Nogier**: investigation (supporting). **Laetitia Vanwonterghem**: investigation (supporting). **Marie‐Carmen Molina**: formal analysis (supporting). **Pau Bernadó**: investigation (supporting); methodology (supporting); validation (supporting); writing—original draft (supporting); writing—review and editing (supporting). **Aurélien Thureau**: investigation (supporting); methodology (supporting); resources (supporting); writing—original draft (supporting). **Jean‐Luc Coll**: conceptualization (supporting); funding acquisition (supporting); supervision (supporting); writing—review and editing (supporting). **Olivier Renaudet**: conceptualization (supporting); funding acquisition (equal); supervision (equal); writing—review and editing (supporting). **Xavier Le Guével**: conceptualization (equal); funding acquisition (equal); supervision (supporting); writing—original draft (equal); writing—review and editing (equal). **Virginie Faure**: conceptualization (lead); funding acquisition (equal); supervision (lead); writing—original draft (lead); writing—review and editing (lead).

## Supporting information

Supplementary Material

## Data Availability

The data that support the findings of this study are available from the corresponding author upon reasonable request.
