# Supplementary material for: Self‐Assembled Peptide‐Gold Nanoclusters with SiRNA Targeting Telomeric Response to Enhance Radiosensitivity in Lung Cancer Cells
Source: Small Sci. 2024 Dec 16;5(2):2400156. doi: 10.1002/smsc.202400156 (PMC11934912; doi:10.1002/smsc.202400156)
Supplement: Supplementary file 1 — Supplementary Material [file SMSC-5-2400156-s001.pdf]

Self-assembled peptide-gold nanoclusters with siRNA targeting telomeric response to enhance radiosensitivity in lung cancer cells.

Sean Moro[1], Mohammed Omrani[1], Sule Erbek[1], Muriel Jourdan[2], Catharina I. Vandekerckhove[1], Cyril Nogier[1], Laetitia Vanwonterghem[1], Marie-Carmen Molina[3], Pau Bernadó[4], Aurélien Thureau[5], Jean-Luc Coll[1]*, Olivier Renaudet[2], Xavier Le Guével[1]*, Virginie Faure[1]*

[1]University Grenoble Alpes, INSERM U1209, CNRS UMR 5309, Institute for Advanced Biosciences, 38000 Grenoble, France

[2]University Grenoble Alpes, CNRS, DCM UMR 5250, F-38000 Grenoble, France

[3]University Grenoble Alpes, CNRS, UMR5063, ICMG FR 2607, Department of Molecular Pharmacochemistry, 38000 Grenoble

[4] Centre de Biologie Structurale (CBS). Université Montpellier, INSERM, CNRS. 29 rue de Navacelles 34090 Montpellier, France

[5] Swing Beamline, Synchrotron SOLEIL, 91192 Gif sur Yvette, France

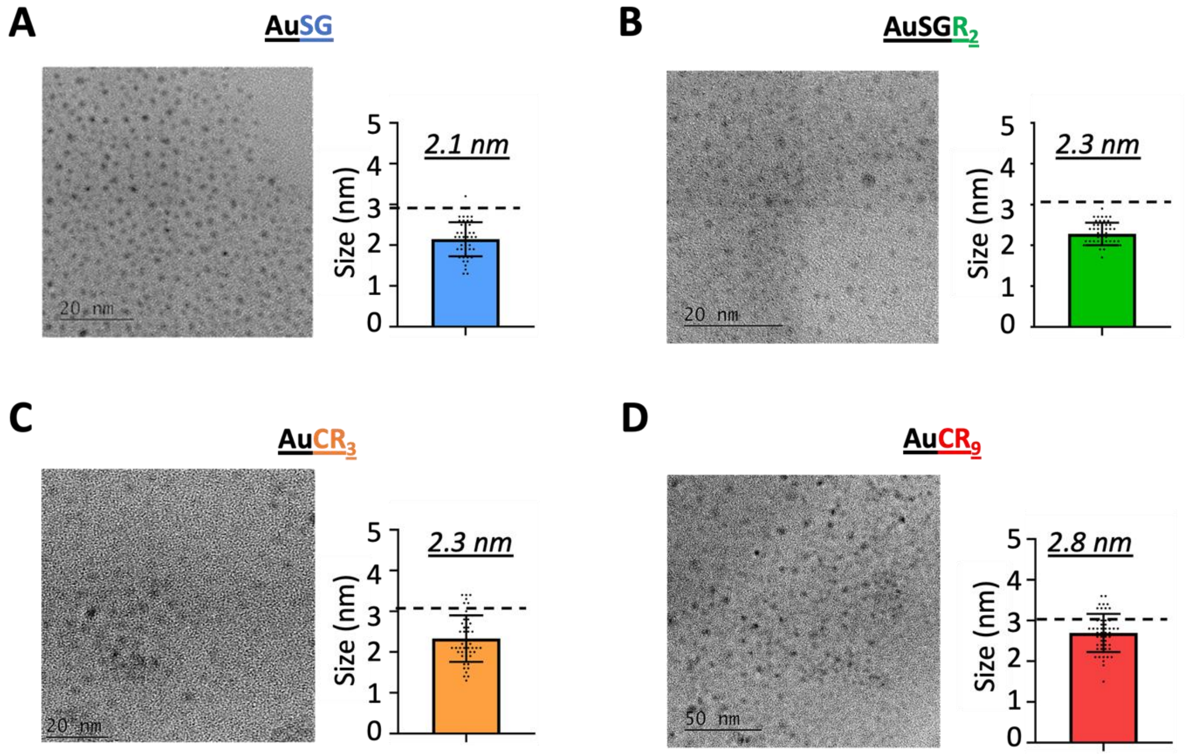

Figure S1. HRTEM images of peptides-AuNCs (**A**) AuSG, (**B**) $AuSGR_2$, (**C**) $AuCR_3$, and (**D**) $AuCR_9$.

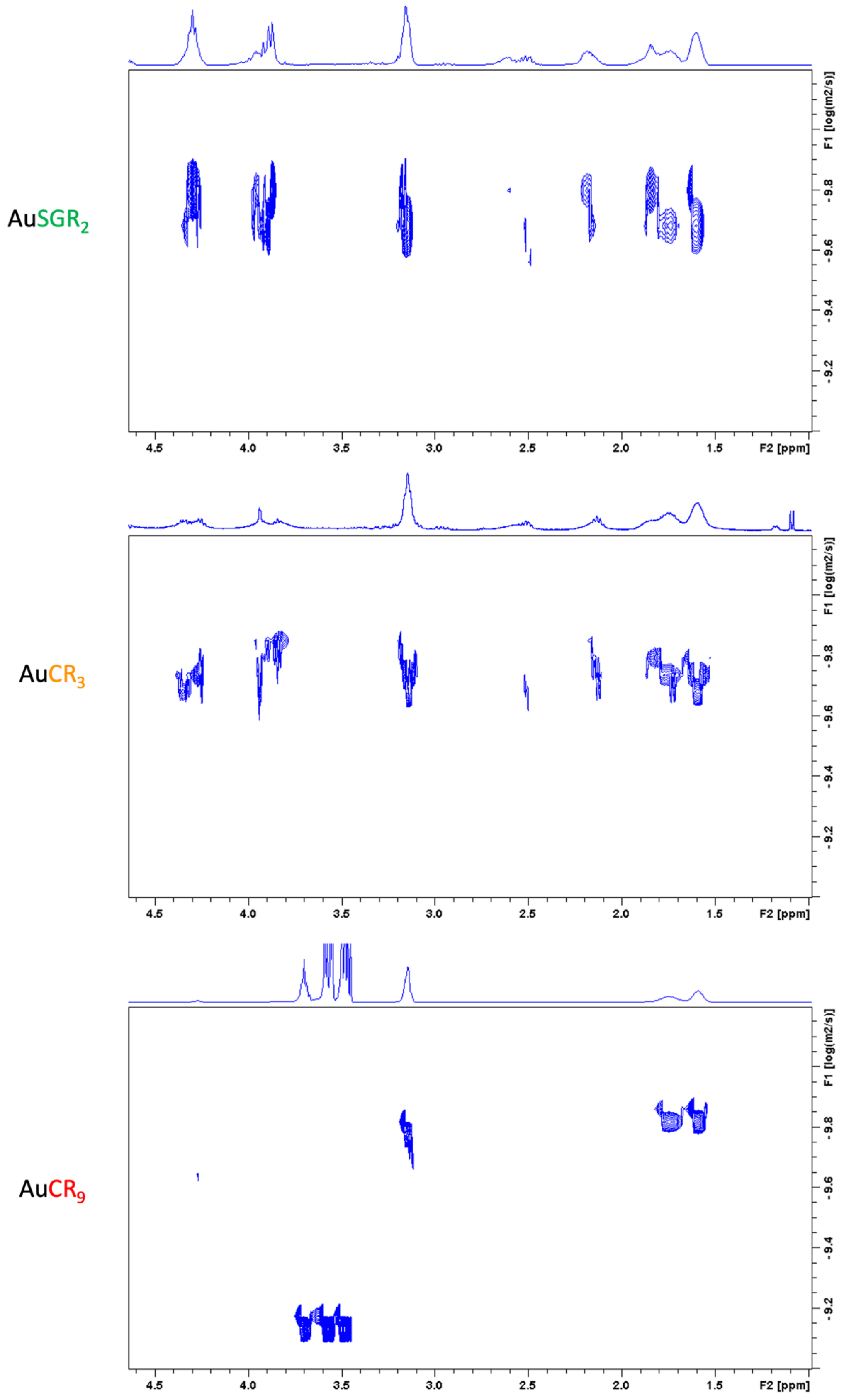

Figure S2. NMR DOSY experiments of $AuSGR_2$, $AuCR_3$, and $AuCR_9$ in D_2O at 25°C.

Glutathione (SG) *(Glu-Cys-Gly)*

Groups	pH			
	< 2,2	< 2,3	7,0	< 9,6
Glu *NH3+/NH2*	+	+	+	0
Glu *COOH/COO-*	0	-	-	-
Gly *COOH/COO-*	0	0	-	-
**Total charge**	**+1**	**0**	**-1**	**-2**

Glutathione modified with 2 arginines (SGR_2) *(Glu-Cys-Gly-Arg-Arg)*

Groups	pH				
	< 2,0	< 2,2	7,0	< 9,6	< 12,1
Glu *NH3+/NH2*	+	+	+	0	0
Glu *COOH/COO-*	0	0	-	-	-
Arg1 *NH3+/NH2*	+	+	+	+	0
Arg2 *NH3+/NH2*	+	+	+	+	0
Arg2 *COOH/COO-*	0	-	-	-	-
**Total charge**	**+3**	**+2**	**+1**	**0**	**-2**

Tetrapeptide (CR_3) *(Cys-Arg-Arg-Arg)*

Groups	pH				
	< 2,0	< 2,2	7,0	< 9,6	< 12,1
Cys *NH3+/NH2*	+	+	+	0	0
Arg1 *NH3+/NH2*	+	+	+	+	0
Arg2 *NH3+/NH2*	+	+	+	+	0
Arg3 *NH3+/NH2*	+	+	+	+	0
Arg3 *COOH/COO-*	0	-	-	-	-
**Total charge**	**+4**	**+3**	**+3**	**+2**	**-1**

Decapeptide (CR_9) *(Cys-Arg-Arg-Arg-Arg-Arg-Arg-Arg-Arg-Arg)*

Groups	pH				
	< 2,0	< 2,2	7,0	< 9,6	< 12,1
Cys *NH3+/NH2*	+	+	+	0	0
Arg1 *NH3+/NH2*	+	+	+	+	0
Arg2 *NH3+/NH2*	+	+	+	+	0
Arg3 *NH3+/NH2*	+	+	+	+	0
Arg4 *NH3+/NH2*	+	+	+	+	0
Arg5 *NH3+/NH2*	+	+	+	+	0
Arg6 *NH3+/NH2*	+	+	+	+	0
Arg7 *NH3+/NH2*	+	+	+	+	0
Arg8 *NH3+/NH2*	+	+	+	+	0
Arg9 *NH3+/NH2*	+	+	+	+	0
Arg9 *COOH/COO-*	0	-	-	-	-
**Total charge**	**+10**	**+9**	**+9**	**+8**	**-1**

Figure S3. Theoretical charge of **SG, SGR_2, CR_3, CR_9** depending on pH.

AuNCs	Ratio SG / R	Estimated number SG / R	Charge SG / R	Global theoritical charge of AuNCs
**AuSG**	100 / 0	18 / 0	-1 / 0	**-18**
**AuSGR$_2$**	_	_	-1 / +2	**+18**
**AuCR$_3$** *(+25% de SG)*	25 / 75	4,5 / 13,5	-1 / +3	**+36**
**AuCR$_9$** *(+25% de SG)*	25 / 75	4,5 / 13,5	-1 / +9	**+117**

Figure S4. Calculation of theoritical charge for each AuNCs synthesized at pH 7. (Example for AuCR$_3$: (-1*4.5) + (3*13.5) = 36). SG: glutathione; R: arginine.

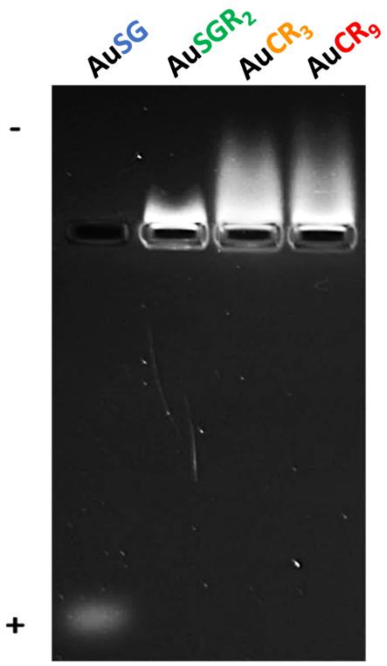

Figure S5. Electrophoretic migration profile of peptide-AuNCs : AuSG, AuSGR$_2$, AuCR$_3$, and AuCR$_9$ in 4% agarose gel in tris-glycine 1X ($\lambda_{exc.}$ 365nm; $\lambda_{em.}$ 705 nm).

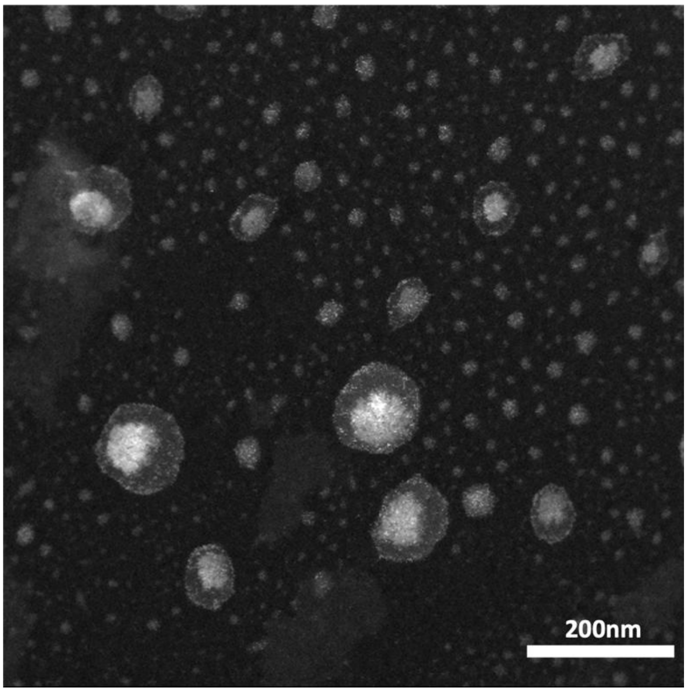

Figure S6. STEM (Scanning Transmission Electron Microscopy) image of the self-assembled $AuSGR_2$-siRNA.

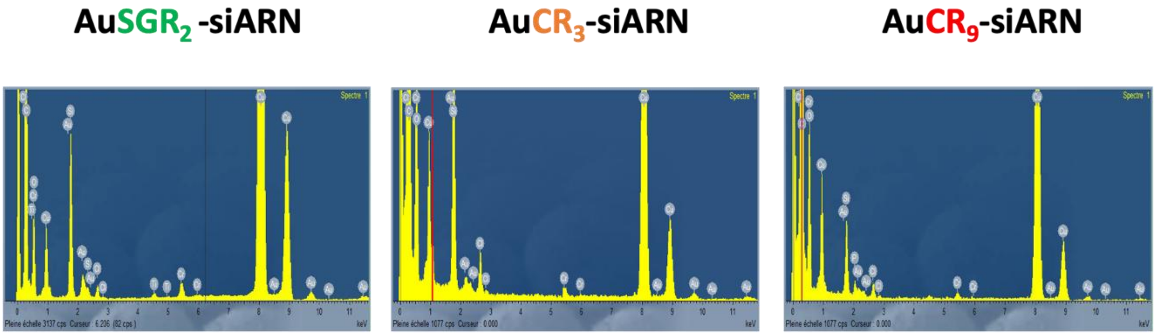

Figure S7. EDS (Energy-Dispersive X-ray Spectroscopy) measurements of the self-assembled AuNCs-siRNA : $AuSGR_2$-siRNA, $AuCR_3$-siRNA, $AuCR_9$-siRNA.

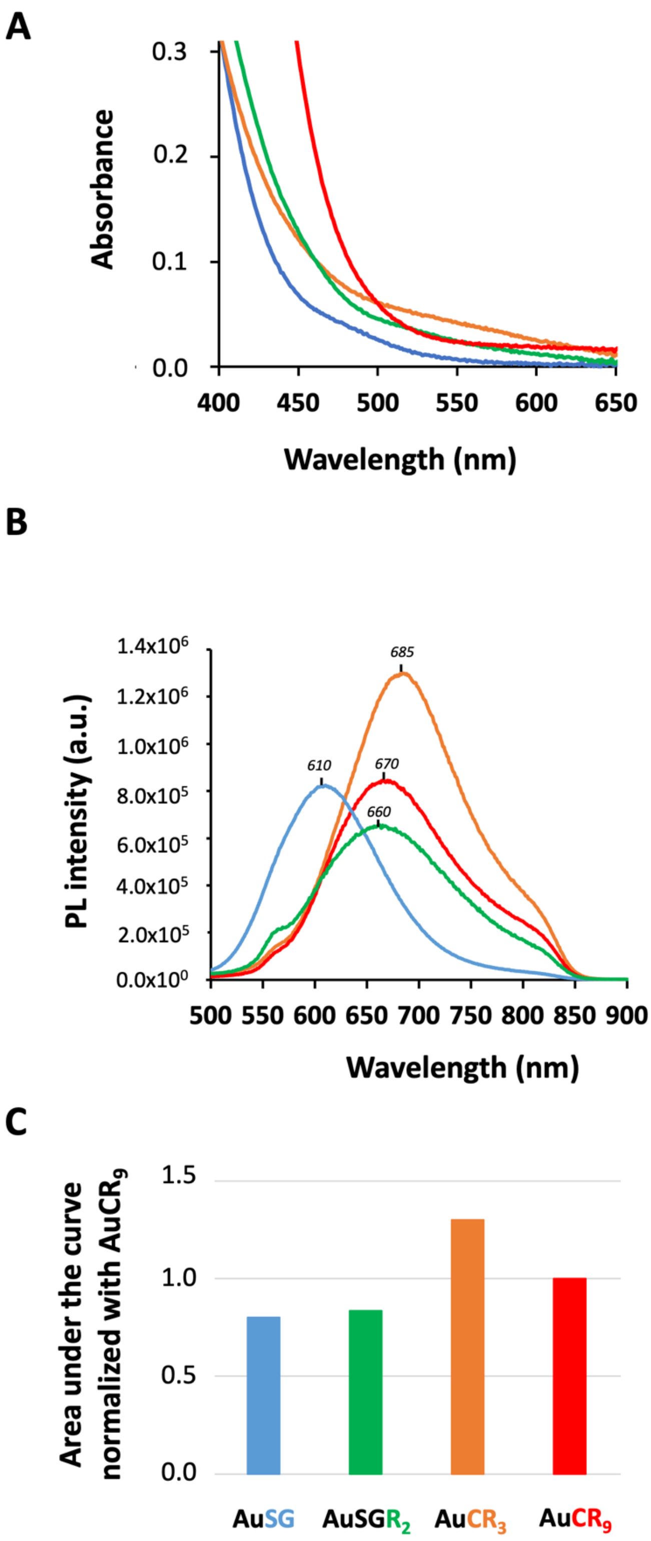

Figure S8. Absorbance spectra (**A**) and PL spectra (**B**) of AuNCs dispersed in PBS pH 7 ($\lambda_{exc.}$ 450 nm). B. inset : PL emission area normalised to $AuCR_9$. (**C**) Area under the curve in (**B**) of AuNCs.

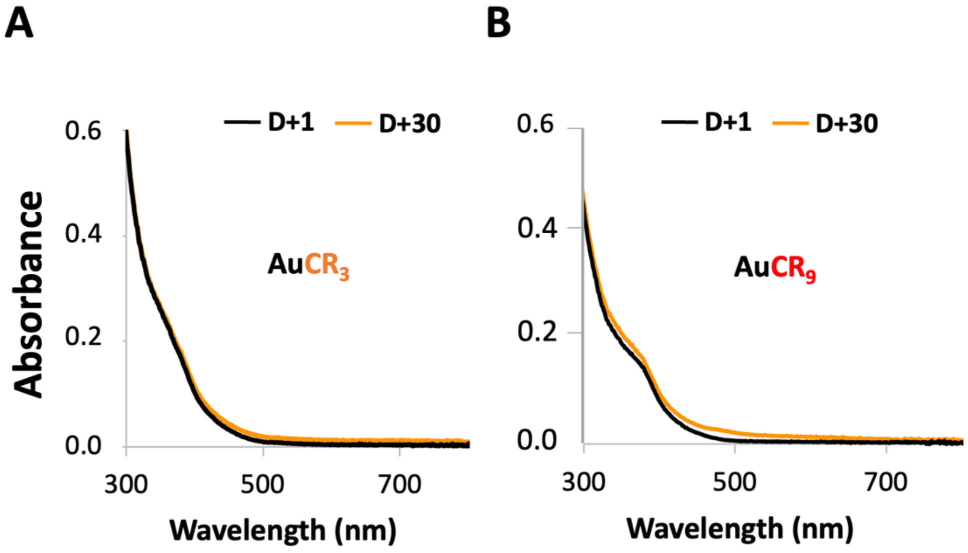

Figure S9. **Stability analysis of AuNCs.** Absorbance spectra of $AuCR_3$ (**A**) and $AuCR_9$ (**B**) dispersed in PBS pH 7 (10 mM) after 1 and 30 days.

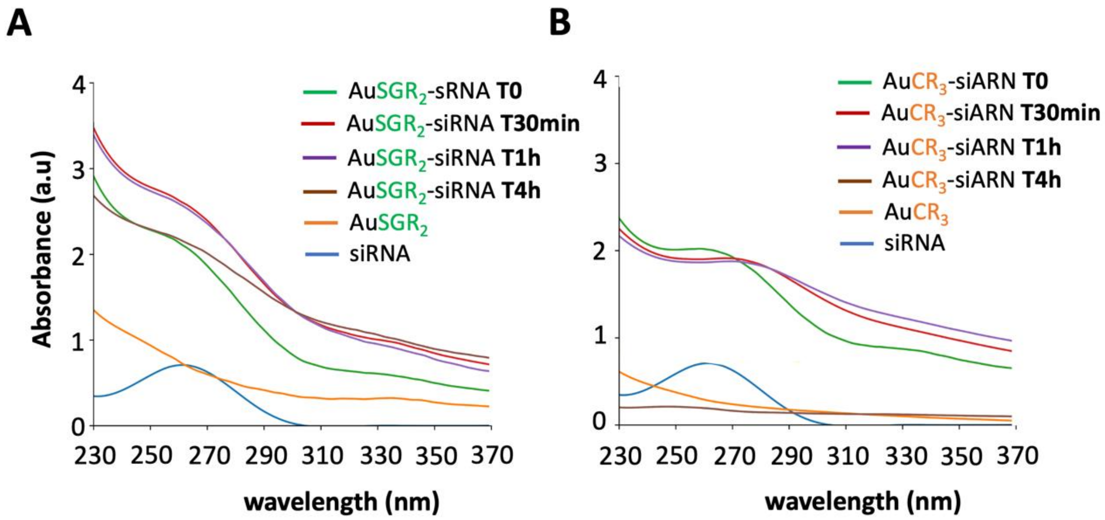

Figure S10. **Stability analysis after self-assembly of AuNCs-siRNA during a kinetic of time.** Absorbance spectra of $AuSGR_2$ complexed or not with siRNA (**A**) and $AuCR_3$ complexed or not with siRNA (**B**) dispersed in PBS pH 7 (10 mM) during a kinetic from 0h until 4h.

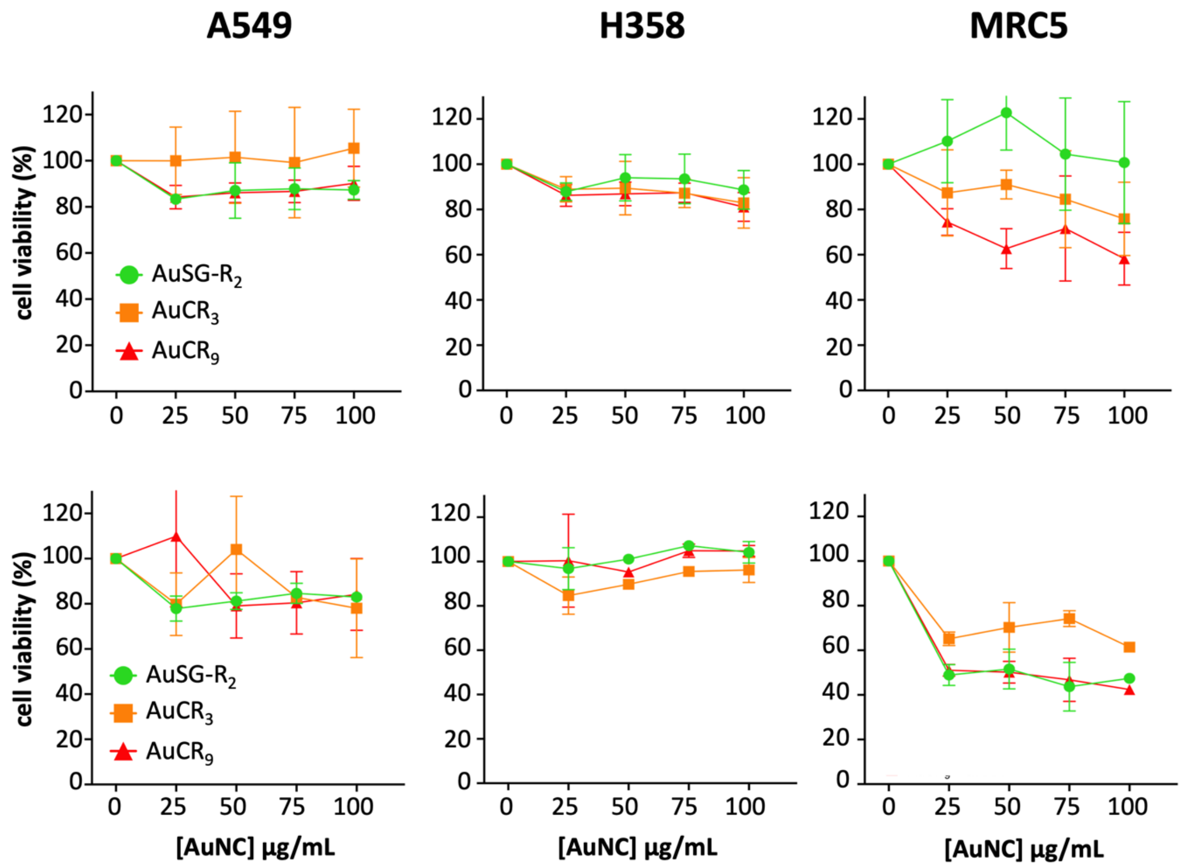

Figure S11. Cytotoxicity analyses of increasing concentrations of AuNCs with or without complexation with siRNA in human lung cancer cell lines (A549, H358) and of AuNCs in human fibroblasts (MRC5-SV2) after 72 hours of incubation using a prestoblue assay.

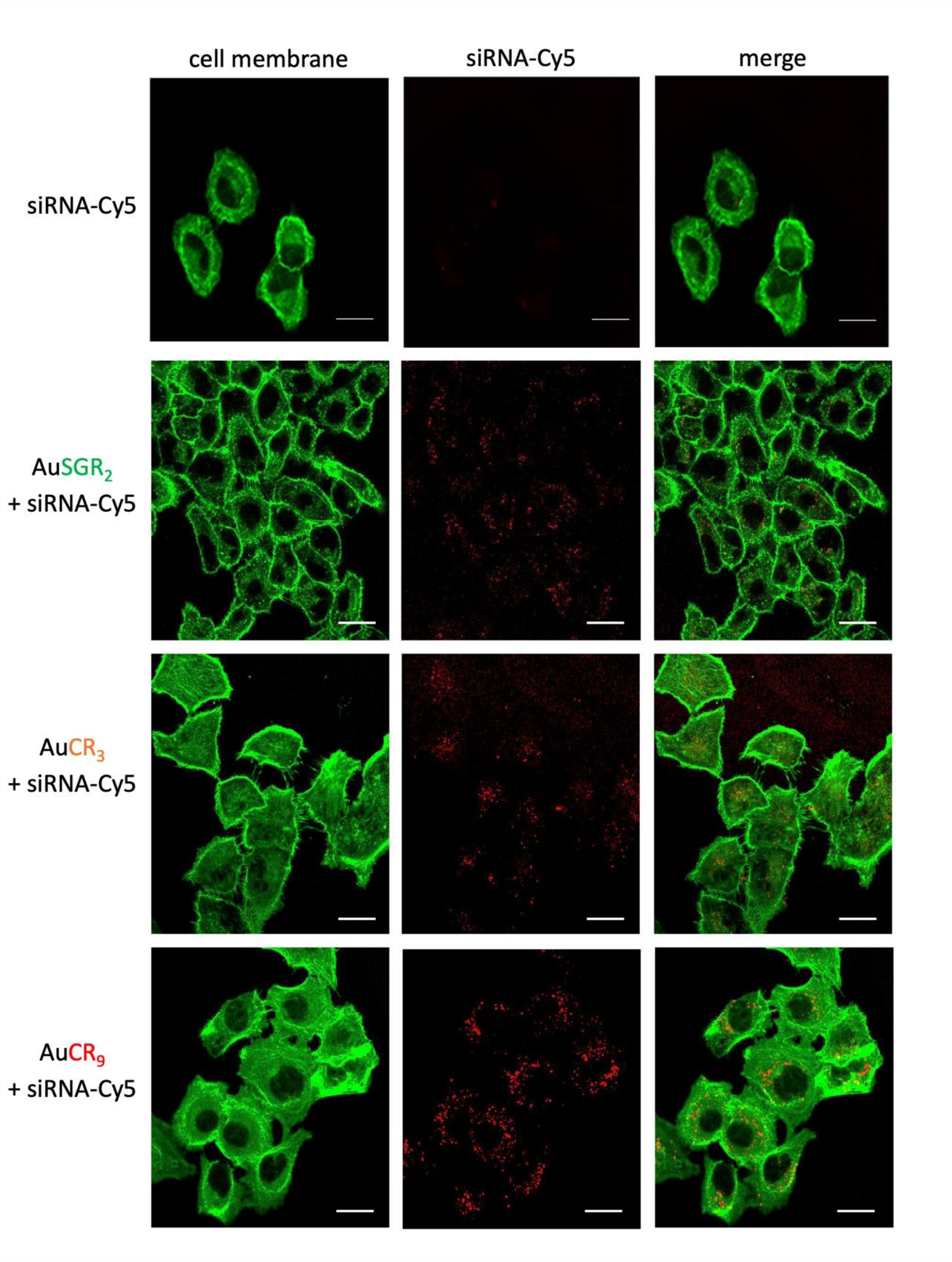

Figure S12. Efficient cellular uptake of AuNCs complexed with siRNA. A549 cells were transfected with cyanine5 (Cy5)-labelled $siRNA_{TRF2}$ free or complexed with AuNCs

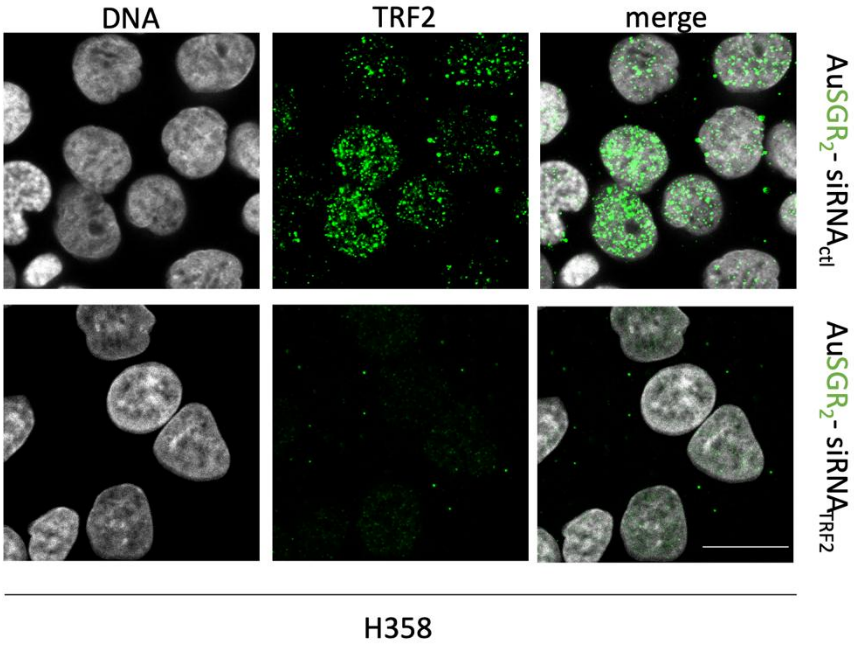

($AuSGR_2$ or $AuCR_3$ or $AuCR_9$). Cell Mask was used to stain the cells. The cellular uptake was monitored by confocal fluorescence microscopy 3 hours post-transfection (scale bar: 20 µm).

Figure S13: Efficient TRF2 downregulation in H358 cells line transfected with $AuSGR_2$-$siRNA_{TRF2}$. Immunofluorescence detection of TRF2 using fluorescence microscopy in cells transfected with the self-assembled $AuSGR_2$-siRNA targeting TRF_2 or a gene control (ctl). DNA was stained with Hoechst. Scale bar corresponds to 10µm.

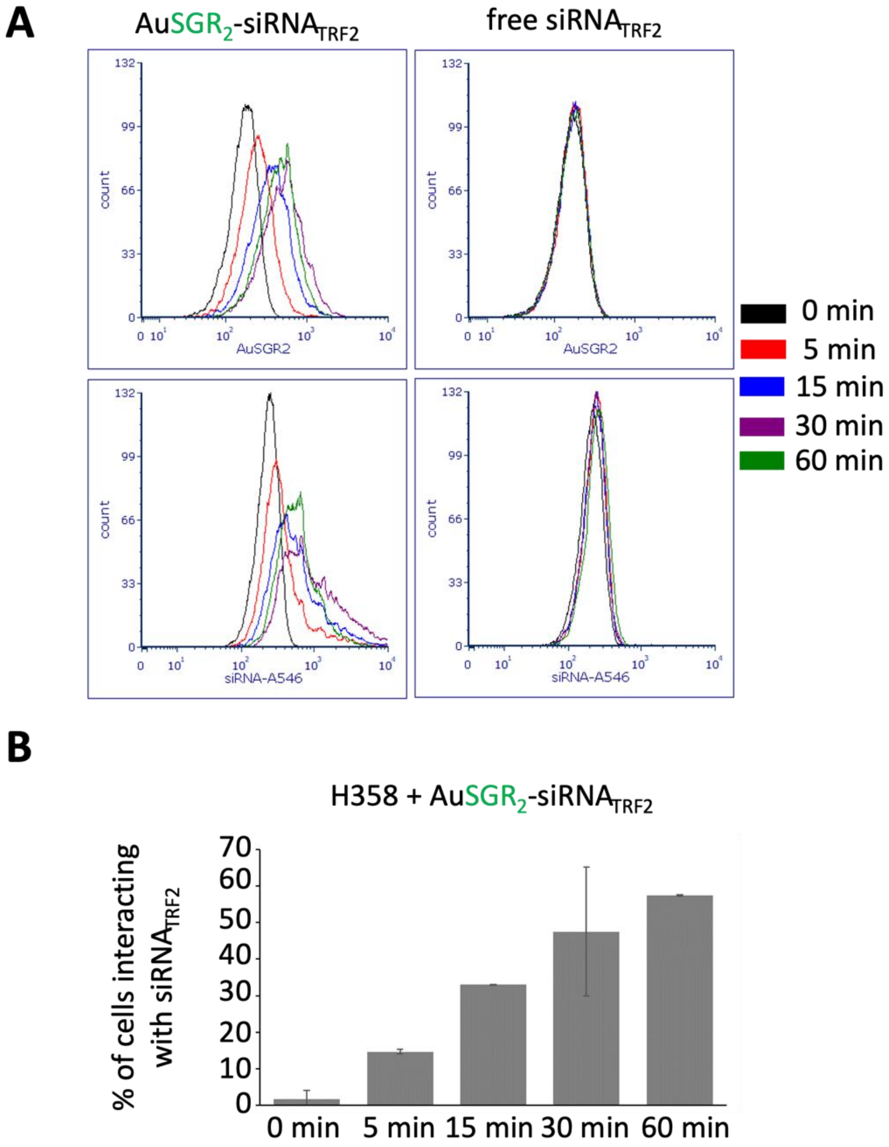

Figure S14. Kinetic of cellular interaction of AuSGR$_2$ complexed with siRNA in H358 cell line. H358 cells were incubated or not for different time periods with A546-labelled siRNA$_{TRF2}$ free or complexed with AuSGR$_2$, analyzed by flow cytometry (**A**) and quantified (**B**) for positive cells interacting with siRNA$_{TRF2}$. (n= 2).

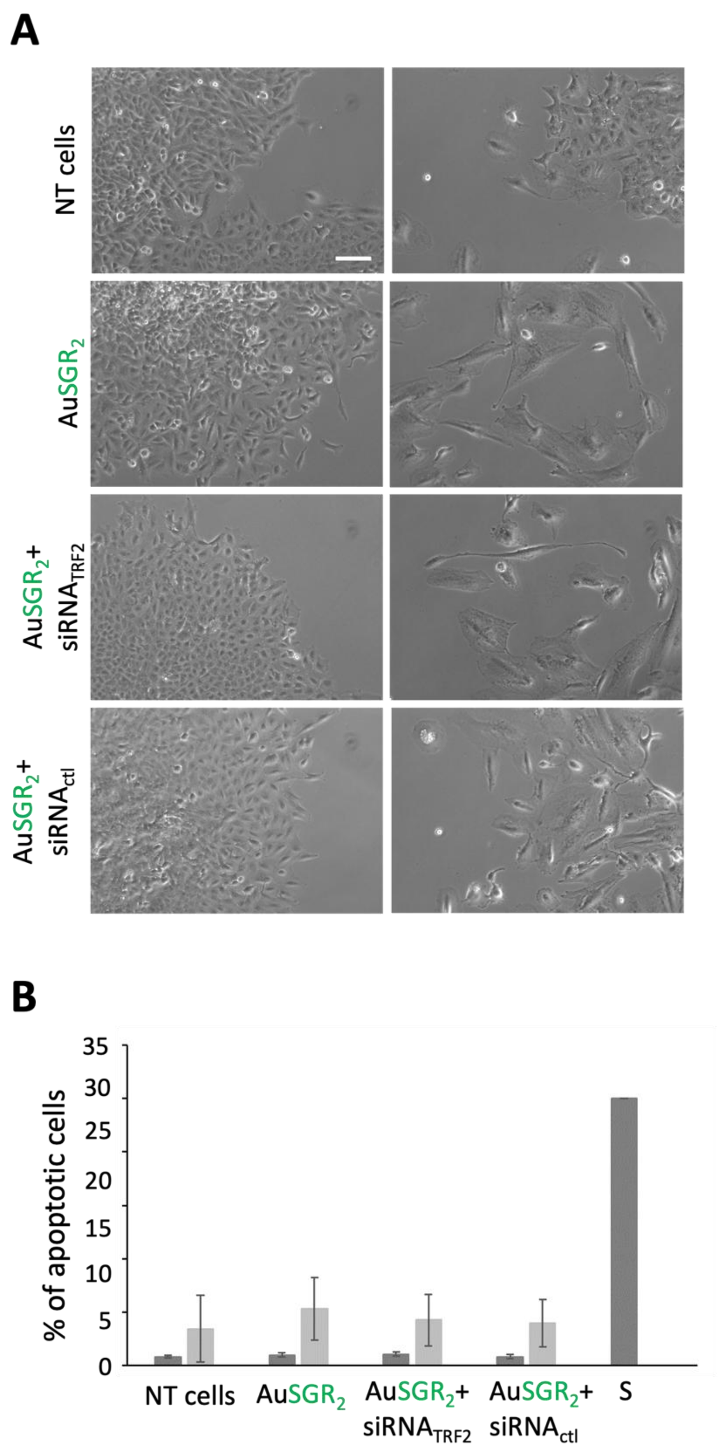

Figure S15. Self-assembled AuSGR$_2$-siRNA$_{TRF2}$ treatment impacts A549 cells morphology after ionizing radiation without inducing cell apoptosis. A. Representative images showing morphology of cells treated or not (NT) with AuSGR$_2$, AuSGR$_2$-siRNA$_{TRF2}$ or AuSGR$_2$-siRNA$_{ctl}$ before (0 Gy) and 96 hours after IR (8 Gy) using a phase contrast microscope. Scale= 100 µm. **B**. Quantification by flow cytometry of the percentage of cells in apoptosis using a caspase3 assay in the different conditions tested. Staurosporin (S) was

used as a positive control of

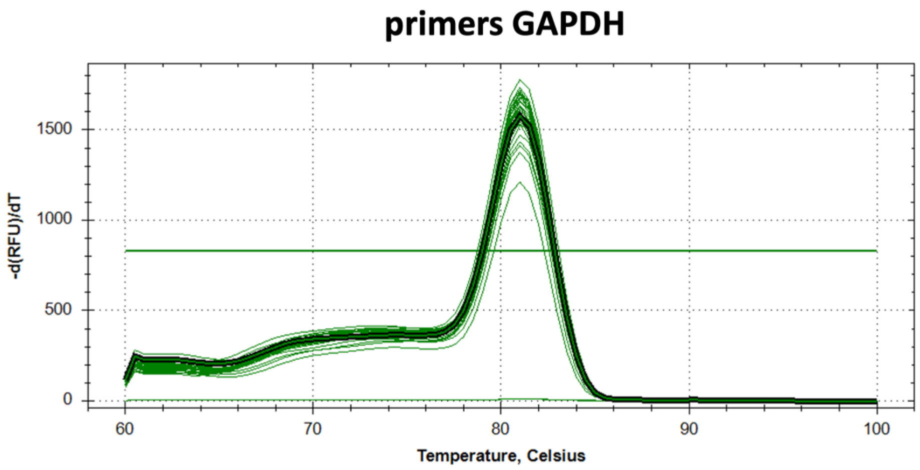

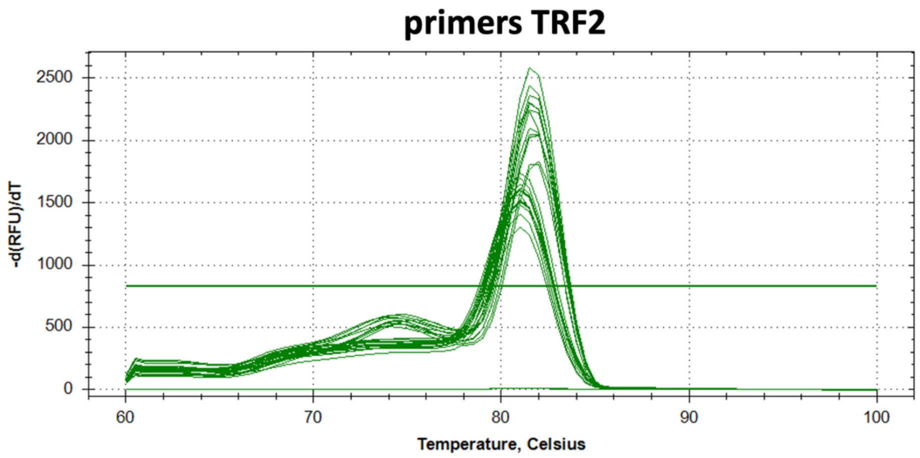

apoptotic cells. The experiment was performed in triplicate (n=3).

Figure S16. Melting peaks of primers used for qPCR**.**
